# Revealing speckle obscured living human retinal cells with artificial intelligence assisted adaptive optics optical coherence tomography

**DOI:** 10.1038/s43856-024-00483-1

**Published:** 2024-04-10

**Authors:** Vineeta Das, Furu Zhang, Andrew J. Bower, Joanne Li, Tao Liu, Nancy Aguilera, Bruno Alvisio, Zhuolin Liu, Daniel X. Hammer, Johnny Tam

**Affiliations:** 1 National Eye Institute, National Institutes of Health, Bethesda, MD 20892 USA; 2 Center for Devices and Radiological Health, U.S. Food and Drug Administration, Silver Spring, MD 20993 USA

**Keywords:** Optical imaging, Retina, Three-dimensional imaging, Interference microscopy

## Abstract

**Background:**

In vivo imaging of the human retina using adaptive optics optical coherence tomography (AO-OCT) has transformed medical imaging by enabling visualization of 3D retinal structures at cellular-scale resolution, including the retinal pigment epithelial (RPE) cells, which are essential for maintaining visual function. However, because noise inherent to the imaging process (e.g., speckle) makes it difficult to visualize RPE cells from a single volume acquisition, a large number of 3D volumes are typically averaged to improve contrast, substantially increasing the acquisition duration and reducing the overall imaging throughput.

**Methods:**

Here, we introduce parallel discriminator generative adversarial network (P-GAN), an artificial intelligence (AI) method designed to recover speckle-obscured cellular features from a single AO-OCT volume, circumventing the need for acquiring a large number of volumes for averaging. The combination of two parallel discriminators in P-GAN provides additional feedback to the generator to more faithfully recover both local and global cellular structures. Imaging data from 8 eyes of 7 participants were used in this study.

**Results:**

We show that P-GAN not only improves RPE cell contrast by 3.5-fold, but also improves the end-to-end time required to visualize RPE cells by 99-fold, thereby enabling large-scale imaging of cells in the living human eye. RPE cell spacing measured across a large set of AI recovered images from 3 participants were in agreement with expected normative ranges.

**Conclusions:**

The results demonstrate the potential of AI assisted imaging in overcoming a key limitation of RPE imaging and making it more accessible in a routine clinical setting.

## Plain language summary

The retinal pigment epithelium (RPE) is a single layer of cells within the eye that is crucial for vision. These cells are unhealthy in many eye diseases, and this can result in vision problems, including blindness. Imaging RPE cells in living human eyes is time consuming and difficult with the current technology. Our method substantially speeds up the process of RPE imaging by incorporating artificial intelligence. This enables larger areas of the eye to be imaged more efficiently. Our method could potentially be used in the future during routine eye tests. This could lead to earlier detection and treatment of eye diseases, and the prevention of some causes of blindness.

## Abstract (web summary)

Das and colleagues develop and evaluate a parallel discriminator generative adversarial network (P-GAN) for improved in-vivo imaging of retinal cellular structures. The P-GAN network improves retinal pigment epithelium contrast 3.5-fold and the overall throughput 99-fold.

## Introduction

High-resolution in vivo ophthalmic imaging enables visualization and quantification of cells^1^, offering the possibility of revealing the status of individual cells in health and disease. For many optical imaging instruments, noise inherent in the imaging processes reduces contrast. The most direct way to suppress noise is the incoherent averaging of a large number of volumes^2–4^. However, this lengthens the overall acquisition time, not only due to the additional volumes required, but also, because of the possibility for artifacts or registration errors across the sequentially acquired volumes due to constant involuntary eye movements that translate and distort the cellular visualization obtained from the microscopic imaging field of view (FOV) (~0.5 mm × 0.5 mm) that is commonly used in adaptive optics (AO) retinal imaging^1,5,6^.

Adaptive optics optical coherence tomography (AO-OCT) is an emerging ophthalmic imaging tool that relies on the detection of interfered light to enable 3D visualization of the retina at single cell level resolution, directly in the living human eye^1,7–9^. However, AO-OCT volumes are inherently susceptible to speckle noise contamination, which arises due to the interference between light scattered from multiple points within the cells^10^. This high contrast, complex intensity distribution of speckle noise can mask cells and limit the visibility of cellular structures. In particular, the retinal pigment epithelial (RPE) cells, which are essential for maintaining visual function^11^ have low intrinsic contrast compared to speckle noise and therefore are challenging to image directly^12^. To overcome the low intrinsic contrast, a large number of AO-OCT volumes need to be averaged (e.g., 120 volumes) in order to visualize the cells^13^. These volumes, obtained by repeatedly imaging the same retinal patch, must be acquired at a sufficiently spaced time interval (about 5 s) to allow for the speckle to decorrelate across volumes^14^. Not only does this strategy substantially increase the total acquisition time, but it also introduces the potential for eye motion artifacts and patient fatigue, both of which can degrade image quality. For some applications, hardware modifications have been proposed to allow for the repeated volume acquisitions to be obtained more quickly, based on shortening the speckle decorrelation time^15,16^ or by producing uncorrelated speckle patterns via frequency^17^, angular^18^, or polarization compounding^19^. However, the capability to visualize cellular details from only a single acquisition alone, rather than from the multiple acquisitions that are still needed even with these hardware modifications, could substantially reduce the time needed to visualize the RPE cells and would be a transformative step towards making AO-OCT a more efficient clinical imaging tool.

Data-driven artificial intelligence (AI) methods have offered promising solutions to generative modeling tasks such as denoising of OCT images^20,21^, high-resolution reconstruction of OCT angiograms^22,23^, and data augmentation in AO images^24^. Here, we explore the potential of AI for recovering the complete cellular structure from only a single noisy AO-OCT acquisition. The deep learning-based generative adversarial network (GAN) provides a powerful framework for synthesizing realistic-looking images from random noise through a competition between discriminator and generator networks^25,26^, and has been successfully applied for image enhancement applications in which a low signal-to-noise ratio (SNR) image is used as input to generate a high SNR counterpart^21,26–28^. Following earlier applications of GAN for improving image quality and training stability^29–31^, conditional GAN (C-GAN)^30^ was introduced to better control the quality of the synthesized images by supplying the discriminator with image and ground truth pairs instead of images alone, as used in the original GAN. However, supervised training of C-GAN with paired images can sometimes be restrictive. Thus, CycleGAN^31^ overcame this requirement by using multiple generator and discriminator networks along with specialized loss functions from unpaired images. Although these strategies have greatly improved image synthesis and style transfer, control over image characteristics have mostly focused on global image features with little or no control over fine local object details, such as individual cells within images. In the case of AO-OCT RPE images which are masked by an overwhelming amount of speckle noise, it is difficult to visualize cellular structure in a single, unaveraged image, making the cell recovery process extremely challenging.

Building upon the various GANs that have been developed, we hypothesize that the generator can better recover cellular structure if the discriminator is enhanced to specifically evaluate similarities in local structural details between the generated and the ground truth averaged images. We describe developing and evaluating a custom GAN framework that contains a generator, a Siamese twin discriminator, as well as a convolutional neural network (CNN) discriminator to recover the RPE cellular structures from single, unaveraged, and noisy AO-OCT images. As both the discriminators work in parallel towards providing strong feedback to the generator network to synthesize perceptually similar images to the ground truth (averaged images), we call the proposed network parallel discriminator GAN (P-GAN). We show that after training, the generator can be applied to recover the cellular morphology from only a single speckled RPE image. This, in turn, enables wide-scale visualization of the RPE mosaic from AO-OCT images acquired across multiple contiguous retinal locations, as the overall time required for RPE visualization at a single location is substantially reduced by eliminating the need for multiple volume acquisition and averaging. The incorporation of AI into the overall image acquisition strategy has the potential to transform the current state-of-the-art ophthalmic imaging with an estimated improvement of 99-fold in the overall throughput.

## Methods

### Adaptive optics imaging

Participants with no history or signs of ocular disease were recruited for this study between the years 2019 to 2022. All participants underwent a comprehensive ophthalmic assessment. In total, eight eyes from seven healthy participants (aged: 29.1 ± 9.1 years) from the National Eye Institute Eye Clinic (National Institutes of Health, Bethesda, Maryland, USA) were imaged using a custom-built AO-OCT retinal imager^13^. Eyes were dilated with 2.5% phenylephrine hydrochloride (Akorn Inc.) and 1% tropicamide (Sandoz, A Novartis Division). This study was approved by the Institutional Review Board of the National Institutes of Health (NCT02317328). Research procedures adhered to the tenets of the Declaration of Helsinki. Written, informed consent was obtained from all participants after the nature of the research and possible consequences of the study were explained.

### Experimental design

#### Data for training and validating AI models

**Fig. 1:**
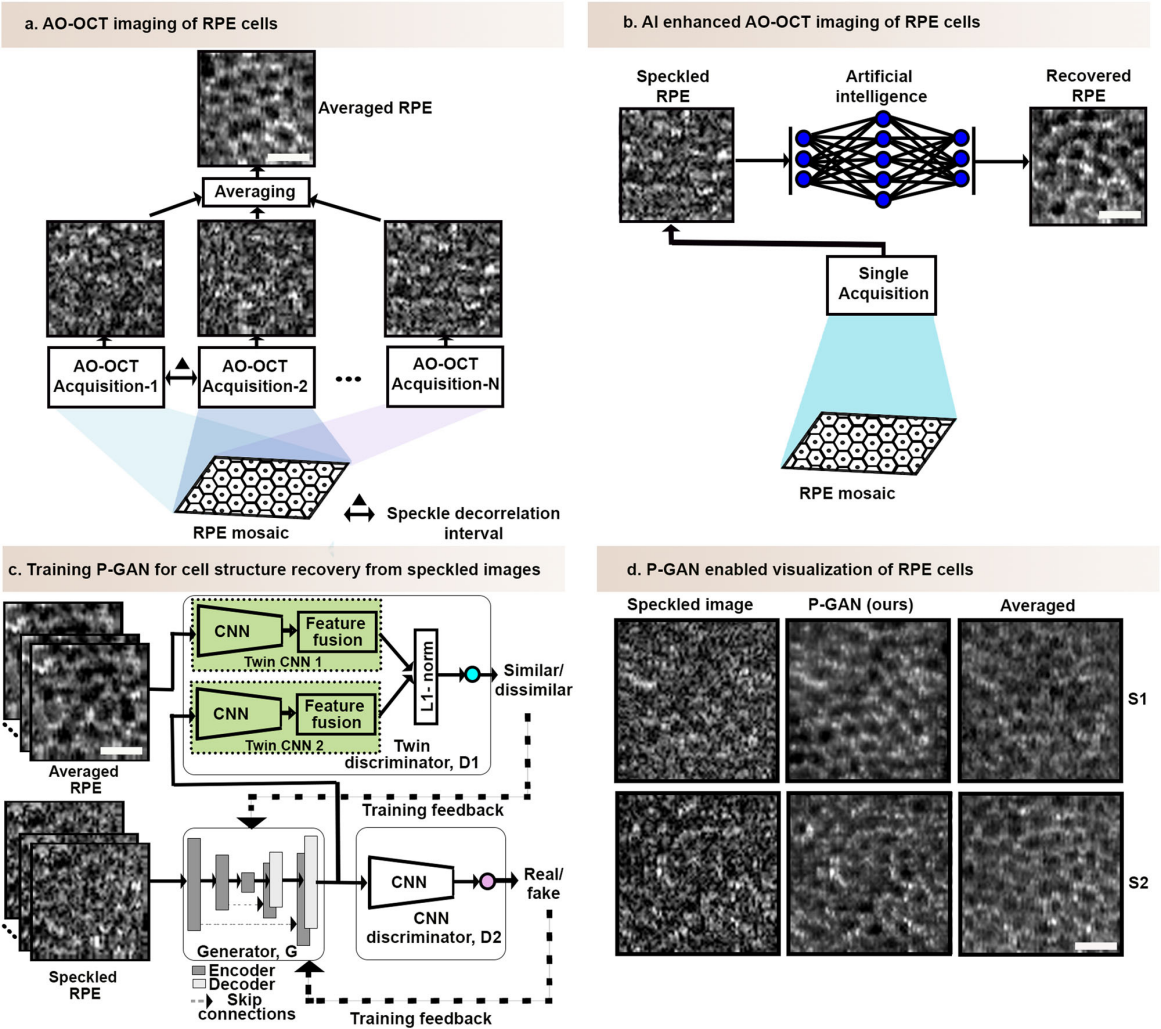
Overview of artificial intelligence (AI) enhanced retinal pigment epithelial (RPE) cell imaging strategy. **a** Adaptive optics optical coherence tomography (AO-OCT) imaging based on multiple acquisitions (120 volumes) at sufficiently spaced time intervals (about 5 s)^13,14^. The en face images of the RPE cells (obtained by segmenting the 2D image of the RPE layer from the 3D volume) have a speckled appearance in a single acquisition but can be averaged across multiple acquisitions to reveal the individual RPE cells appearing as the dark cell centers with bright cell surroundings in the averaged en face image. **b** AO-OCT imaging enhanced with AI can recover the cellular features from only a single speckled AO-OCT acquisition, thereby eliminating the need for multiple volume acquisition for averaging and substantially reducing the imaging duration. **c** Parallel discriminator generative adversarial network (P-GAN), the proposed AI model, is comprised of three networks: a generator (G) to recover the cellular structures from the speckled images of the RPE cells, a twin discriminator (D1) with two identical twin convolutional neural networks (CNNs) to perform a one-to-one feature comparison of the recovered images from the generator and the averaged (ground truth) images yielding a similarity score, and a CNN discriminator (D2) that assigns a label of fake/real to the recovered images. The adversarial learning of the three networks facilitates the faithful recovery of both local structural details as well as the global mosaic of the RPE cells. Matched speckled and averaged image pairs are used to train P-GAN. Details about the network architecture are presented in Supplementary Fig. 2. After training, the trained generator can be deployed to reveal the cellular details from speckled images obtained from a single AO-OCT acquisition. **d** Applying the trained generator to predict the cellular structures of the RPE cells of two participants (S1 and S2) from the corresponding speckled images. The ground truth averaged images (average of 120 acquired AO-OCT volumes) are shown for comparison. Scale bar: 25 µm.

AO-OCT volumes from five eyes of five participants were acquired at a rate of 147 kHz (300 × 300 pixels at a rate of 1.6 volumes per second) from up to four retinal locations ranging from 0–3 mm temporal to the fovea with a FOV of 1.5 degrees. At each location, 120 speckled volumes were acquired. Following image acquisition, volumes were digitally flattened based on the outer retinal layers, corrected for eye motion after manual selection of reference frames for registration, and then averaged to generate ground truth averaged RPE en face images^13^ (Fig. 1a and Supplementary Fig. 1). Due to a scanner artifact (distortion arising from the turnaround of the scanner from one line to the next), 50 pixels from the left and right sides of the image were cropped off to yield a final image of 300 × 200 pixels. The reference frames (speckled images) and the ground truth averaged images are used as training data for the AI model in a supervised manner.

#### Experimental data for RPE assessment from the recovered images

For the large-scale assessment of the RPE cells, speckled volumes from 63 overlapping locations spanning a 1 mm × 3 mm region of the retina from the fovea extending in the temporal direction were acquired from three participants (Supplementary Table 1). To ensure that the algorithm performance was assessed on never-seen images, three eyes that were not used in the training and validation of the AI model were selected for this purpose. To facilitate image acquisition and to allow for brief breaks in between acquisitions, a total of 10 volumes were acquired at each location, from which the one with the least distortion (subjectively determined minimal motion artifacts and no eye blinks) was selected as input to the P-GAN for cellular recovery. To validate the accuracy of RPE recovery on the experimental data, additional volumes (120 volumes) were acquired from four retinal locations of the three participants. The ground truth averaged images were created by averaging 120 speckled volumes for objective image recovery comparison.

#### Model details

The proposed framework (P-GAN) contains a generator (G), a siamese twin discriminator (D1), as well as a CNN discriminator (D2) (Fig. 1c and Supplementary Fig. 2). G takes the specked image as input and creates an image of the RPE using a series of CNN-based encoder and decoder network components (Supplementary Fig. 2 and P-GAN network architecture in Supplementary Methods). D1 is designed to use Siamese twin neural network^32^, which has a specialized architecture (Supplementary Fig. 2) to naturally rank similarity between the generator created and averaged RPE images in a representative feature space using L1 norm^33^. We found through experimentation that fusing features from two intermediate layers with the last convolutional layer of the twin network with appropriate weights ensured better cellular recovery (Supplementary Tables 2, 3). We introduced a weighted feature fusion (WFF) block that concatenated features from three layers of the twin network to estimate the similarity (Supplementary Fig. 2 and P-GAN network architecture in Supplementary Methods). Additionally, D2 also helped the cell recovery process by determining if the images recovered by G were closer to the statistical distribution of the averaged ground truth images. G, D1, and D2 were simultaneously trained using two adversarial and content loss functions (Objective loss functions in Supplementary Methods).

The dataset to train the model was created from the acquired volumes from five participants. The training dataset was augmented by leveraging the natural eye motion of the participants during imaging by selecting multiple (up to 50) reference (speckled) frames to create a set of ground truth averaged images that were each slightly shifted with respect to each other (Supplementary Fig. 3). In addition, due to the combination of simultaneously occurring eye motion and point-scanning nature of image acquisition, each of the averaged images also contained unique intravolume distortions, which served as a further means for natural data augmentation. This resulted in a total of 5968 image patches (150 × 150 pixels) extracted from the speckled and averaged image pairs used for training. P-GAN was trained for 100 epochs with a batch size of 8 using an Adam optimizer with a learning rate of 0.0002, and exponential decay rates of 0.5 for the first moment and 0.9 for the second moment. Four NVIDIA TITAN V graphical processing units (GPU) were used to accelerate the training process. After training is complete, the discriminators were no longer needed, and the generator could be used to recover the RPE structure from the speckled images (Fig. 1d).

To evaluate the performance, a leave-one-participant-out validation protocol was used. A total of forty paired images at different retinal locations from the five participants were used for validation of the method. Four objective image quality assessment metrics (Validation metrics in Supplementary Methods): perceptual image error assessment through pairwise preference (PieAPP)^34^, learned perceptual image patch similarity (LPIPS)^35^, deep image structure and texture similarity (DISTS)^36^, and Fréchet Inception Distance (FID)^37^ were used to validate the performance.

#### Quantification of cellular spacing and contrast

Cell spacing and contrast were quantified to assess the efficacy of P-GAN for RPE recovery. Cell spacing was estimated using the circumferentially averaged power spectrum^38^ of each image region of interest (200 × 200 pixels). The peak spatial frequency of the spectrum (i.e., the RPE fundamental frequency) was an estimate of cell spacing. To convert from pixels to µm, a paraxial ray trace on a three-surfaced simplified model eye^39^ was used after replacing the axial length, corneal curvature, and anterior chamber depth with measurements of these values obtained from each participant (IOL Master, Carl Zeiss Meditec)^40^.

Voronoi neighborhoods were generated from the manually identified cell centers on selected images to analyze the packing properties of the RPE cells. At least two expert graders sequentially marked each image and then interactively re-reviewed images until full consensus on the markings were achieved. The cellular contrast of the P-GAN-created images and the averaged images were compared using a peak distinctiveness measure, defined as the height of the peak in the circumferentially averaged power spectrum computed as the difference between the log power spectral density (PSD) between the peak and the local minima to the left of the peak.

### Reporting summary

Further information on research design is available in the Nature Portfolio Reporting Summary linked to this article.

## Results

### P-GAN enables visualization of cellular structure from a single speckled image

The overall goal was to learn a mapping between the single speckled and averaged images (Fig. 1b) using a paired training dataset. Inspired by the ability of traditional GAN networks to recover aspects of the cellular structure (Supplementary Fig. 4), we sought to further improve upon these networks with P-GAN. In our network architecture (Supplementary Fig. 2), the twin and the CNN discriminators were designed to ensure that the generator faithfully recovered both the local structural details of the individual cells as well as the overall global mosaic of the RPE cells. In addition, we incorporated a WFF strategy to the twin discriminator that concatenated features from different layers of the twin CNN with appropriate weights, facilitating effective comparisons and learning of the complex cellular structures and global patterns of the images.

P-GAN was successful in recovering the retinal cellular structure from the speckled images (Fig. 1d and Supplementary Movie 1). Toggling between the averaged RPE images (obtained by averaging 120 acquired AO-OCT volumes) and the P-GAN recovered images showed similarity in the cellular structure (Supplementary Movie 2). Qualitatively, P-GAN showed better cell recovery capability than other competitive deep learning networks (U-Net^41^, GAN^25^, Pix2Pix^30^, CycleGAN^31^, medical image translation using GAN (MedGAN)^42^, and uncertainty guided progressive GAN (UP-GAN)^43^) (additional details about network architectures and training are shown in Other network architectures section in Supplementary Methods and Supplementary Table 4, respectively) with clearer visualization of the dark cell centers and bright cell surroundings of the RPE cells (e.g., magenta arrows in Supplementary Fig. 4 and Supplementary Movie 3), possibly due to the twin discriminator’s similarity assessment. Notably, CycleGAN was able to generate some cells that were perceptually similar to the averaged images, but in certain areas, undesirable artifacts were introduced (e.g., the yellow circle in Supplementary Fig. 4).

Quantitative comparison between P-GAN and the off-the-shelf networks (U-Net^41^, GAN^25^, Pix2Pix^30^, CycleGAN^31^, MedGAN^42^, and UP-GAN^43^) using objective performance metrics (PieAPP^34^, LPIPS^35^, DISTS^36^, and FID^37^) further corroborated our findings on the performance of P-GAN (Supplementary Table 5). There was an average reduction of at least 16.8% in PieAPP and 7.3% in LPIPS for P-GAN compared to the other networks, indicating improved perceptual similarity of P-GAN recovered images with the averaged images. Likewise, P-GAN also achieved the best DISTS and FID scores among all networks, demonstrating better structural and textural correlations between the recovered and the ground truth averaged images. Overall, these results indicated that P-GAN outperformed existing AI-based methods and could be used to successfully recover cellular structure from speckled images.

### Twin discriminator improves cell recovery performance

**Fig. 2:**
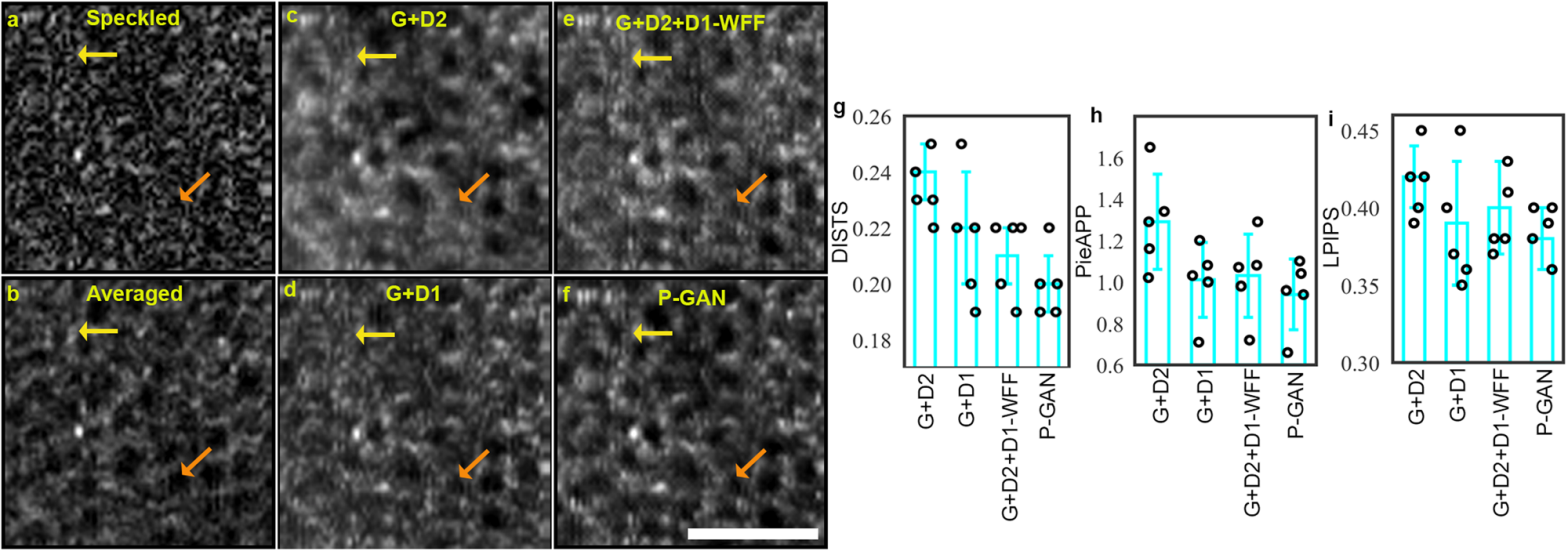
Effect of parallel discriminator generative adversarial network (P-GAN) components on the recovery of retinal pigment epithelial (RPE) cells. **a** Single speckled image compared to images of the RPE obtained via **b** average of 120 volumes (ground truth), **c** generator with the convolutional neural network (CNN) discriminator (G + D2), **d** generator with the twin discriminator (G + D1), **e** generator with CNN and twin discriminators without the weighted feature fusion (WFF) module (G + D2 + D1-WFF), and **f** P-GAN. The yellow and orange arrows indicate cells that are better visualized using P-GAN compared to the intermediate models. **g–i** Comparison of the recovery performance using deep image structure and texture similarity (DISTS), perceptual image error assessment through pairwise preference (PieAPP), and learned perceptual image patch similarity (LPIPS) metrics. The bar graphs indicate the average values of the metrics across sample size, *n* = 5 healthy participants (shown in circles) for different methods. The error bars denote the standard deviation. Scale bar: 50 µm.

Our preliminary explorations of the off-the-shelf GAN frameworks showed that these methods have the potential for recovering cellular structure and contrast but alone are insufficient to recover the fine local cellular details in extremely noisy conditions (Supplementary Fig. 4). To further reveal and validate the contribution of the twin discriminator, we trained a series of intermediate models and observed the cell recovery outcomes. We began by training a conventional GAN, comprising of the generator, G, and the CNN discriminator, D2. Although GAN (G + D2) showed promising RPE visualization (Fig. 2c) relative to the speckled images (Fig. 2a), the individual cells were hard to discern in certain areas (yellow and orange arrows in Fig. 2c). To improve the cellular visualization, we replaced D2 with the twin discriminator, D1. Indeed, a 7.7% reduction in DISTS was observed with clear improvements in the visualization of some of the cells (orange arrows in Fig. 2c, d).

Having shown the outcomes of training D1 and D2 independently with G, we showed that combining both D1 and D2 with G (P-GAN) boosted the performance even further, evident in the improved values (lower scores implying better perceptual similarity) of the perceptual measures (Fig. 2g–i). For this combination of D1 and D2, we replaced the WFF block, which concatenated features from different layers of the twin CNN with appropriate weights, with global average pooling of the last convolutional layer (G + D2 + D1-WFF). Without the WFF, the model did not adequately extract powerful discriminative features for similarity assessment and hence resulted in poor cell recovery performance. This was observed both qualitatively (yellow and orange arrows in Fig. 2e, f) as well as quantitatively with the higher objective scores (indicating low perceptual similarity with ground truth averaged images) for G + D2 + D1-WFF compared to P-GAN (Fig. 2g–i).

Taken together, this established that the CNN discriminator (D2) helped to ensure that recovered images were closer to the statistical distribution of the averaged images, while the twin discriminator (D1), working in conjunction with D2, ensured structural similarity of local cellular details between the recovered and the averaged images. The adversarial learning of G with D1 and D2 ensured that the recovered images not only have global similarity to the averaged images but also share nearly identical local features.

Finally, experimentation using different weighting configurations in WFF revealed that the fusion of the intermediate layers with weights of 0.2 with the last convolutional layer proved complementary in extracting shape and texture information for improved performance (Supplementary Tables 2, 3). These ablation experiments indicated that the global perceptual closeness (offered by D2) and the local feature similarity (offered by D1 and WFF) were both important for faithful cell recovery.

### Leveraging eye motion for data augmentation

Given the relatively recent demonstration of RPE imaging using AO-OCT in 2016^12^, and the long durations needed to generate these images, currently, there are no publicly available datasets for image analysis. Therefore, we acquired a small dataset using our custom-built AO-OCT imager^13^ consisting of seventeen retinal locations obtained by imaging up to four different retinal locations for each of the five participants (Supplementary Table 1). To obtain this dataset, a total of 84 h was needed (~2 h for image acquisition followed by 82 hours of data processing which included conversion of raw data to 3D volumes and correction for eye motion-induced artifacts). After performing traditional augmentation (horizontal flipping), this resulted in an initial dataset of only 136 speckled and averaged image pairs. However, considering that this and all other existing AO-OCT datasets that we are aware of are insufficient in size compared to the training datasets available for other imaging modalities^44,45^, it was not surprising that P-GAN trained on this initial dataset yielded very low objective perceptual similarity (indicated by the high scores of DISTS, PieAPP, LPIPS, and FID in Supplementary Table 6) between the recovered and the averaged images.

To overcome this limitation, we leveraged the natural eye motion of the participants to augment the initial training dataset. The involuntary fixational eye movements, which are typically faster than the imaging speed of our AO-OCT system (1.6 volumes/s), resulted in two types of motion-induced artifacts. First, due to bulk tissue motion, a displacement of up to hundreds of cells between acquired volumes could be observed. This enabled us to create averaged images of different retinal locations containing slightly different cells within each image. Second, due to the point-scanning nature of the AO-OCT system compounded by the presence of continually occurring eye motion, each volume contained unique intra-frame distortions. The unique pattern of the shifts in the volumes was desirable for creating slightly different averaged images, without losing the fidelity of the cellular information (Supplementary Fig. 3). By selecting a large number of distinct reference volumes onto which the remaining volumes were registered, we were able to create a dataset containing 2984 image pairs (22-fold augmentation compared to the initial limited dataset) which was further augmented by an additional factor of two using horizontal flipping, resulting in a final training dataset of 5996 image pairs for P-GAN (also described in Data for training and validating AI models in Methods). Using the augmented dataset for training P-GAN yielded high perceptual similarity of the recovered and the ground truth averaged images which was further corroborated by improved quantitative metrics (Supplementary Table 6). By leveraging eye motion for data augmentation, we were able to obtain a sufficiently large training dataset from a recently introduced imaging technology to enable P-GAN to generalize well for never-seen experimental data (Supplementary Table 1 and Experimental data for RPE assessment from the recovered images in Methods).

### Objective assessment of the cellular contrast offered by AI

**Fig. 3:**
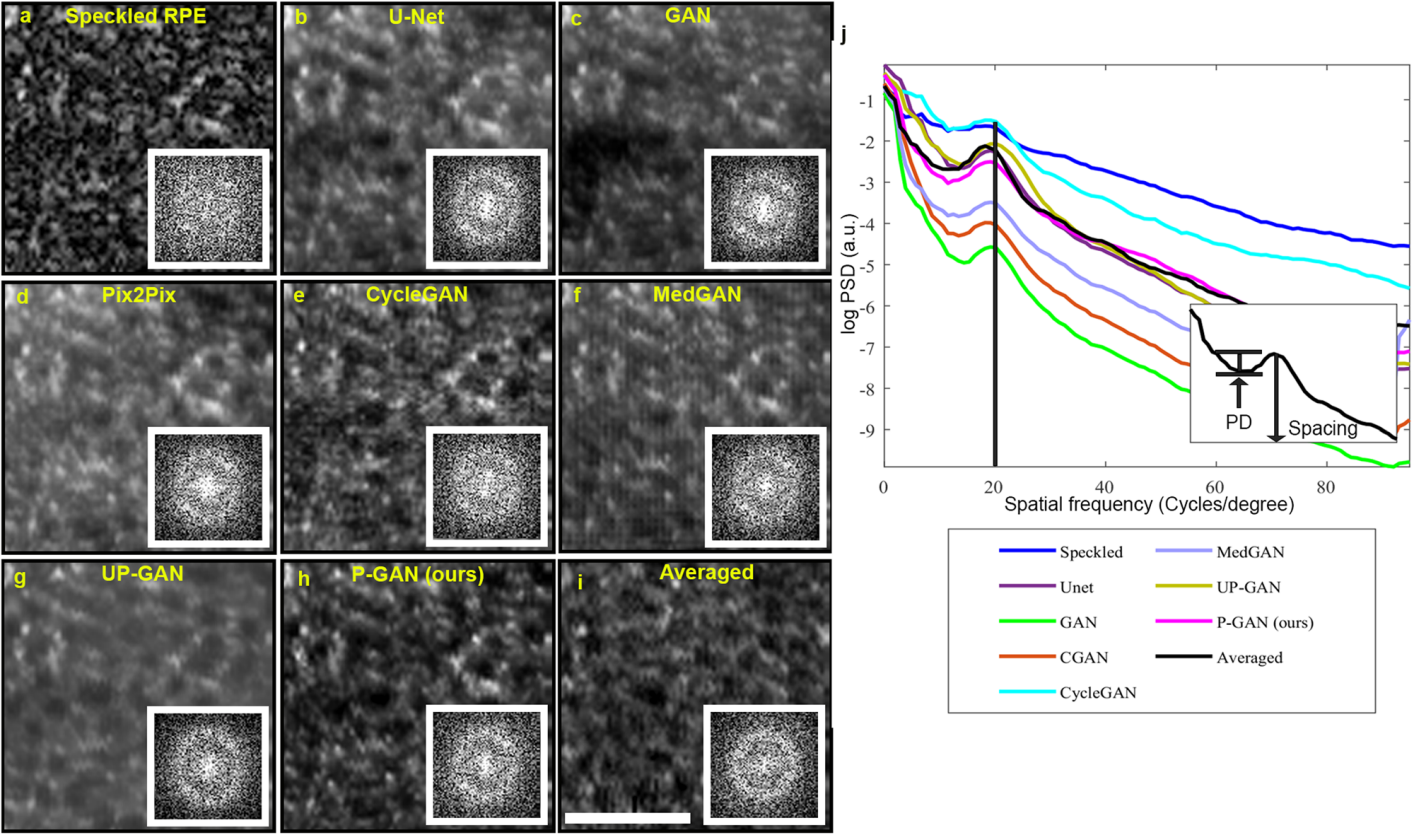
Using power spectra analysis to estimate the cellular contrast achieved using artificial intelligence (AI). **a** Example specked image acquired from participant S1. Recovered images using **b** U-Net, **c** generative adversarial network (GAN), **d** Pix2Pix, **e** CycleGAN, **f** medical image translation using GAN (MedGAN), **g** uncertainty guided progressive GAN (UP-GAN), **h** parallel discriminator GAN (P-GAN). **i** Ground truth averaged image (obtained by averaging 120 adaptive optics optical coherence tomography (AO-OCT) volumes). Insets in (**a–i**) show the corresponding 2D power spectra of the images. A bright ring representing the fundamental spatial frequency of the retinal pigment epithelial (RPE) cells can be observed in U-Net, GAN, Pix2Pix, CycleGAN, MedGAN, UP-GAN, P-GAN, and averaged images power spectrum corresponds to the cell spacing. **j** Circumferentially averaged power spectral density (PSD) for each of the images. A visible peak corresponding to the RPE cell spacing was observed for U-Net, GAN, Pix2Pix, CycleGAN, MedGAN, UP-GAN, P-GAN, and averaged images. The vertical line indicates the approximate location of the fundamental spatial frequency associated with the RPE cell spacing. The height of the peak (defined as peak distinctiveness (PD)) indicates the RPE cellular contrast measured as the difference in the log PSD between the peak and the local minima to the left of the peak (inset in (**j**)). Scale bar: 50 µm.

In addition to the structural and perceptual similarity that we demonstrated between P-GAN recovered and averaged images, here, we objectively assessed the degree to which cellular contrast was enhanced by P-GAN compared to averaged images and other AI methods. As expected, examination of the 2D power spectra of the images revealed a bright ring in the power spectra (indicative of the fundamental spatial frequency present within the healthy RPE mosaic arising from the regularly repeating pattern of individual RPE cells) for the recovered and averaged images (insets in Fig. 3b–i).

Interestingly, although this ring was not readily apparent on the speckled single image (inset in Fig. 3a), it was present in all the recovered images, reinforcing our observation of the potential of AI to decipher the true pattern of the RPE mosaic from the speckled images. Furthermore, the radius of the ring, representative of the approximate cell spacing (computed from the peak frequency of the circumferentially averaged PSD) (Quantification of cell spacing and contrast in Methods), showed consistency among the different methods (shown by the black vertical line along the peak of the circumferentially averaged PSD in Fig. 3j and Table 1), indicating high fidelity of recovered cells in comparison to the averaged images.

**Table 1:** Comparison of cellular contrast and cell spacing error across the different networks

Image/Network	Peak distinctiveness (a.u.)	Cell spacing error (µm)
Speckled image	0.13 ± 0.06	-
U-Net	0.36 ± 0.09	−0.9 ± 1.4
GAN	0.39 ± 0.06	−1.0 ± 1.3
Pix2Pix	0.39 ± 0.08	−1.0 ± 1.3
CycleGAN	0.40 ± 0.08	−0.5 ± 1.1
MedGAN	0.34 ± 0.07	−1.1 ± 1.4
UP-GAN	0.45 ± 0.08	−0.9 ± 1.2
P-GAN (ours)	0.46 ± 0.07	−0.9 ± 1.3
Averaged image	0.54 ± 0.09	-

The cell spacing error for specked images is not shown, as there is no visible peak in the power spectral density from which to compute the spacing. The cell spacing errors for the AI methods are computed with respect to the averaged (ground truth) images. All values are expressed as mean ± SD.

The height of the local peak of the circumferentially averaged power spectra (which we defined as peak distinctiveness) provided an opportunity to objectively quantify the degree to which cellular contrast was enhanced. Among the different AI methods, the peak distinctiveness achieved by P-GAN was closest to the averaged images with a minimal absolute error of 0.08 compared to ~0.16 for the other methods (Table 1), which agrees with our earlier results indicating the improved performance of P-GAN. In particular, P-GAN achieved a contrast enhancement of 3.54-fold over the speckled images (0.46 for P-GAN compared with 0.13 for the speckled images). These observations demonstrate P-GAN’s effectiveness in boosting cellular contrast in addition to structural and perceptual similarity.

### AI enables efficient visualization of the RPE mosaic across retinal locations

Having demonstrated the efficacy and reliability of P-GAN on test data, we wanted to evaluate the performance of P-GAN on experimental data from never-seen human eyes across an experimental dataset (Supplementary Table 1), which to the best of our knowledge, covered the largest extent of AO-OCT imaged RPE cells reported (63 overlapping locations per eye). This feat was made possible using the AI-enhanced AO-OCT approach developed and validated in this paper. Using the P-GAN approach, in our hands, it took 30 min of time (including time needed for rest breaks) to acquire single volume acquisitions from 63 separate retinal locations compared to only 4 non-overlapping locations imaged with nearly the same duration using the repeated averaging process (15.8-fold increase in number of locations). Scaling up the averaging approach from 4 to 63 locations would have required nearly 6 h to acquire the same amount of RPE data (note that this does not include any data processing time), which is not readily achievable in clinical practice. This fundamental limitation explains why AO-OCT RPE imaging is currently performed only on a small number of retinal locations^12,13^.

**Fig. 4:**
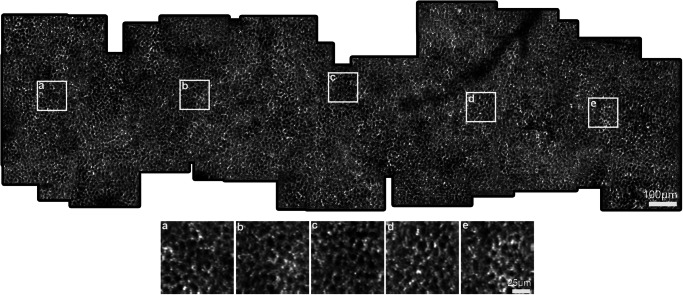
Parallel discriminator generative adversarial network (P-GAN) enabled wide-scale visualization of the retinal pigment epithelial (RPE) cellular mosaic. The image shows the visualization of the RPE mosaic using the P-GAN recovered images (this montage was manually constructed from up to 63 overlapping recovered RPE images from the left eye of participant S2). The white squares (**a**–**e**) indicate regions that are further magnified for better visualization at retinal locations **a** 0.3 mm, **b** 0.8 mm, **c** 1.3 mm, **d** 1.7 mm, and **e** 2.4 mm temporal to the fovea, respectively. Additional examples of montages from two additional participants are shown in Supplementary Fig. 5.

**Fig. 5:**
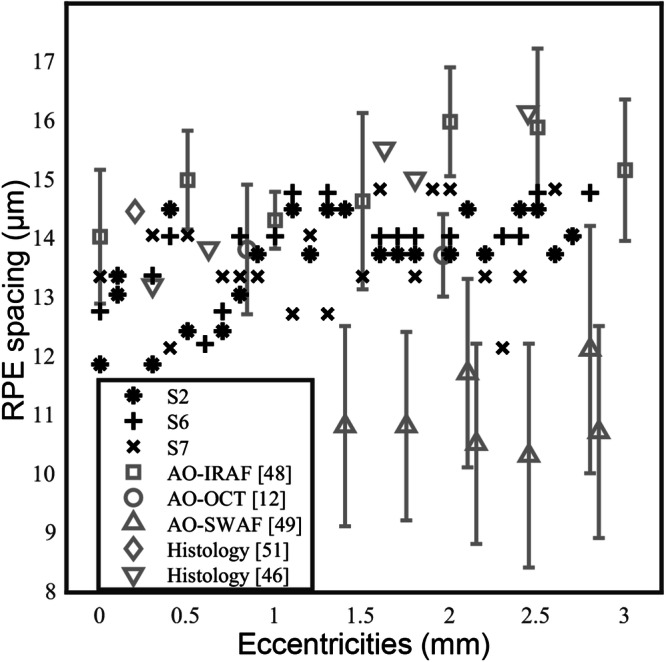
Comparison of cell spacing of the parallel discriminator generative adversarial network (P-GAN) recovered images with previously published data across retinal locations (eccentricities) temporal to the fovea. Symbols in black indicate cell spacing estimated from P-GAN recovered images for three participants (S2, S6, and S7) at different retinal locations. For comparison, data in gray denote the mean and standard deviation values from previously published studies (adaptive optics infrared autofluorescence (AO-IRAF)^48^, adaptive optics optical coherence tomography (AO-OCT)^12^, adaptive optics with short-wavelength autofluorescence (AO-SWAF)^49^, and histology^46,51^).

Leveraging P-GAN’s ability to successfully recover cellular structures from never-seen experimental data, we stitched together overlapping recovered RPE images to construct montages of the RPE mosaic (Fig. 4 and Supplementary Fig. 5). To further validate the accuracy of the recovered RPE images, we also created ground truth averaged images by acquiring 120 volumes from four of these locations per eye (12 locations total) (Experimental data for RPE assessment from the recovered images in Methods). The AI-enhanced and averaged images for the experimental data at the 12 locations were similar in appearance (Supplementary Fig. 6). Objective assessment using PieAPP, DISTS, LPIPS, and FID also showed good agreement with the averaged images (shown by comparable objective scores for experimental data in Supplementary Table 7 and test data in Supplementary Table 5) at these locations, confirming our previous results and illustrating the reliability of performing RPE recovery for other non-seen locations as well (P-GAN was trained using images obtained from up to 4 retinal locations across all participants). The cell spacing estimated using the circumferentially averaged PSD between the recovered and the averaged images (Supplementary Fig. 7 and Supplementary Table 8) at the 12 locations showed an error of 0.6 ± 1.1 µm (mean ± SD). We further compared the RPE cell spacing from the montages of the recovered RPE from the three participants (S2, S6, and S7) with the previously published in vivo studies (obtained using different imaging modalities) and histological values (Fig. 5)^12,46–51^. Considering the range of values in Fig. 5, the metric exhibited inter-participant variability, with cell spacing varying up to 0.5 µm across participants at any given retinal location. Nevertheless, overall our measurements were within the expected range compared to the published normative data^12,46–51^. Finally, peak distinctiveness computed at 12 retinal locations of the montages demonstrated similar or better performance of P-GAN compared to the averaged images in improving the cellular contrast (Supplementary Table 8).

Voronoi analysis performed on P-GAN and averaged images at 12 locations (Supplementary Fig. 8) resulted in similar shapes and sizes of the Voronoi neighborhoods. Cell spacing computed from the Voronoi analysis (Supplementary Table 9) fell within the expected ranges and showed an average error of 0.5 ± 0.9 µm. These experimental results demonstrate the possibility of using AI to transform the way in which AO-OCT is used to visualize and quantitatively assess the contiguous RPE mosaic across different retinal locations directly in the living human eye.

## Discussion

We demonstrated that P-GAN can effectively recover the cellular structure from speckle-obscured AO-OCT images of the RPE. The key feature of our approach is that cellular contrast can be improved using only a single speckled acquisition, completely bypassing the need for sequential volume averaging currently being used for AO-OCT RPE imaging^12,13^. This is an important step towards more routine clinical application of AO-OCT imaging for probing the health of the retinal tissue at the cellular level, especially for the task of morphometric measurements of cell structure across different retinal locations (Figs. 4, 5 and Supplementary Fig. 5).

The success of cellular recovery using P-GAN can be attributed to the Siamese network-inspired twin discriminator that provided local structural cues of feature similarity (between the recovered and the ground truth averaged images) to the generator. The improvement of P-GAN over U-Net, traditional GAN, Pix2Pix, CycleGAN, MedGAN, and UPGAN (Supplementary Fig. 4 and Supplementary Table 5) was unsurprising given that these other networks were not intended to handle highly speckled noisy environments in which the cellular structures were not readily apparent. Our ablation studies indicated the synergistic improvement realized through the WFF combination to the twin discriminator (D1) for the recovery of the fine local structural details and the traditional CNN-based discriminator (D2) for global feature recovery (Fig. 2). In terms of computational complexity, it should also be noted that the network architecture of P-GAN has much fewer number of parameters (8.8-fold) compared to CycleGAN (Supplementary Table 10).

Substantial time saving was realized using our P-GAN-inspired approach, allowing us to cover more than 15-fold more imaging locations in nearly the same amount of imaging time. Without accounting for the possibility of participant fatigue, we estimate that it would have required at least 6 h to acquire the same amount of RPE data (12-fold reduction using P-GAN), illustrating how the integration of AI into the overall image acquisition pipeline can enable novel experimental design of imaging sequences (so as not to relegate AI to only the post-processing regime). On top of the time spent on image acquisition, it must be noted that data handling after image acquisition is an order of magnitude more costly than the image acquisition itself. In our current AO-OCT imager^13^, which acquires streams of raw data at a rate of 640 MB/s (expected to increase substantially with technological advancements), a typical scanning session quickly adds up to terabytes of data for a single participant because of the requirements of averaging (Supplementary Table 11). With P-GAN enabling recovery of the cellular features from a single acquired AO-OCT volume, a 12-fold reduction (2.8 TB for averaging compared to 0.23 TB using P-GAN) in the size of the raw data was achieved. Post-processing of this data to correct for eye motion and other artifacts requires intense computational resources and the processing time for a typical scanning session (four locations with averaging) is on the order of one day or more. The post-processing for 63 locations imaged with repeated averaging would have taken an estimated 13 days as opposed to only 2.7 h using the strategy of cellular recovery from a single acquired volume using P-GAN (116-fold reduction). Overall, considering both the image acquisition time as well as post-processing time, the generation of a 63 location montage was achieved with a substantial time savings of 99-fold.

This paper contributes to the growing trend of using AI for improving spatial or temporal resolution and enhancing SNR in the fields of biomedical imaging^20,52,53^ and biological microscopy^54–56^, especially in the area of speckle noise. Unlike other sources of noise, speckle noise is particularly troublesome to handle due to its complex nature, non-Gaussian distribution, and multiplicative nature^57^ (as opposed to additive noise). Consequently, although promising, traditional approaches suitable for the removal of more classical types of noise did not perform as well as P-GAN. In the case of RPE imaging, there was no visible evidence of cellular structure in single volumes due to the overwhelming presence of speckle noise. Given our demonstrated success in applying P-GAN to this problem, we anticipate the possibility of applying AI to other applications affected by speckle noise in which averaging of sequentially acquired volumes is essential, such as AO-OCT imaging of the transparent inner retinal cells (e.g., ganglion cells)^58^ and optical coherence tomography angiography (OCTA)^59–62^.

Future application of our approach to diseased eyes will first require consensus on image interpretation of diseased RPE which can have substantial differences in contrast, appearance, and size when compared to healthy RPE cells^63^. Also, images of diseased RPE cells will need to be captured in an appropriately sized training dataset. As it is generally more challenging to obtain high-quality images from patients with disease, due in part to the limited amount of clinic time that may be available for assessment, we are hopeful that future improvements using AI-assisted AO imaging will be transformative. Nonetheless, establishing a larger normative database of healthy RPE images is a critical step for comparison with diseased eyes.

In conclusion, we introduced an AI-assisted strategy to enhance the visualization of the cellular details from a single speckle-obscured AO-OCT image that can potentially transform the way in which imaging data is acquired. Not only does this strategy enable the wide-scale visualization and noninvasive assessment of cellular structure in the living human eye, but also, it substantially reduces the time and burden of data handling associated with obtaining data. These advances help to make AO imaging more accessible for routine clinical application and are critical steps towards clarifying our understanding of the structure, function, and pathophysiology of blinding retinal diseases.

## Supplementary information

Supplementary Information

Description of Additional Supplementary Files

Supplementary Movie 1

Supplementary Movie 2

Supplementary Movie 3

Supplementary Data 1

Reporting Summary

## Data Availability

Datasets used for training and validation are not publicly available due to their containing information that could compromise the privacy of research participants. Requests to access the training and validation datasets should be directed to the corresponding author. It may be possible to make data available as part of a future academic collaboration through institutional collaboration agreements and additional IRB approval.

## Code availability

A TensorFlow implementation of P-GAN is publicly available in the Zenodo repository (10.5281/zenodo.10455740).
