# Supplementary material for: Revealing speckle obscured living human retinal cells with artificial intelligence assisted adaptive optics optical coherence tomography
Source: Commun Med (Lond). 2024 Apr 10;4:68. doi: 10.1038/s43856-024-00483-1 (PMC11006674; doi:10.1038/s43856-024-00483-1)
Supplement: Supplementary file 1 — Supplementary Information [file 43856_2024_483_MOESM1_ESM.pdf]

Supplementary Information

Revealing speckle obscured living human retinal cells with artificial intelligence assisted adaptive optics optical coherence tomography

Vineeta Das[1], Furu Zhang[1], Andrew J. Bower[1], Joanne Li[1], Tao Liu[1], Nancy Aguilera[1], Bruno Alvisio[1], Zhuolin Liu[2], Daniel X. Hammer[2], and Johnny Tam[1]

[1]National Eye Institute, National Institutes of Health, Bethesda, MD 20892, USA
[2]Center for Devices and Radiological Health, U.S. Food and Drug Administration, Silver Spring, MD 20993, USA

Supplementary Methods

P-GAN network architecture

P-GAN consists of a generator (G), a twin discriminator (D1), and a CNN discriminator (D2) (**Supplementary Fig. 2**). The generator learns a mapping $G: x \rightarrow \hat{y}$ where x is the speckled image and $\hat{y}$ is the recovered RPE image. The twin discriminator assigns a similarity score by comparing the local structural features of the recovered image from the generator and the ground truth averaged image y. The CNN discriminator compares the overall image distribution between recovered images from the generator and averaged images. G, D1, and D2 are trained simultaneously by optimizing the adversarial and the content objective functions.

***Generator, G**:* The generator uses the U-Net architecture that has previously shown to be successful for various image based applications [1]. The network is composed of an encoding path, a decoding path, and skip connections from the encoder to the decoder modules. In the encoding path, there are five encoder blocks and a convolutional layer with rectified linear unit (ReLU) activation. Each encoder block consists of a convolutional layer, followed by batch normalization (BN) and leaky rectified linear unit (LReLU) activation. The convolution layers in the encoding path have a kernel size (k) of 4 with varying numbers of filters (n) and strides (s) of 2. In the decoding path, there are five decoder blocks, each of which contains a nearest neighbor interpolation block, a convolutional layer followed by BN and LReLU. Concatenation (concat) in the decoder module act as skip connections to link low-level features and high-level features by concatenating their feature maps. In the end, a transpose convolution with Tanh activation is used.

***Twin discriminator, D1**:* The twin discriminator contains two identical twin CNNs to extract features from the image generated by the generator and the averaged images. The twin CNNs have a sequence of four convolutional layers and the weighted feature fusion (WFF) block. The convolution layers have filters of varying sizes and s=1. The kernel sizes follow $7 \times 7, 5 \times 5$, and 3×3, respectively. The number of convolution filters is specified as multiples of 16. Each convolutional layer is followed by ReLU activation and max-pooling layer with filter size of 2 and s= 2. The feature fusion block consists of global average pooling (GAP) layers to summarize the feature maps from different convolutional layers and sigmoid activation for normalization. The features are weighted with empirically designed weights ($\alpha_1 = 1, \alpha_2 = 0.2$, and $\alpha_3 = 0.2$) and are concatenated to form a weighted feature vector. The L1 norm between feature vectors from the twin CNNs is provided to a dense layer with sigmoid activation to obtain the similarity scores.

***CNN discriminator, D2**:* The CNN discriminator consists of a sequence of three convolutional layers, each of which is followed by BN (except for the first layer) and LReLU. The convolution layers have k=4 and s=2 with n specified as multiples of 16. The convolutional layers are followed by GAP, dense and sigmoid activation to obtain labels of fake/real.

Objective loss functions

The twin discriminator, D1 classifies the recovered and the ground truth averaged image pairs $(y, \hat{y})$ as dissimilar and the averaged image pair (y, y) as similar. The generator is forced to generate images that have features similar to the averaged images in order to fool D1. The training of G against D1 forms the adversarial part of the objective and is given as

$$\min_{G} \max_{D1} L_t(G, D1) = E_y[\log D1\,(y, y)] + E_{\hat{y}}[log(1 - D1(y, \hat{y}))] \quad (1)$$

The CNN discriminator, D2 takes the recovered image $\hat{y}$ as input and classifies it as fake while the averaged images y are classified as real. This feedback provided to the generator allows it to generate perceptually superior images, making it increasingly difficult for D2 to correctly discriminate. The adversarial objective for training G and D2 is given as

$$\min_{G} \max_{D2} L_c(G, D2) = E_y[log\, D2\,(y)] + E_{\hat{y}}[log(1 - D2(\hat{y}))] \quad (2)$$

To ensure that the information content is retained in the images recovered by the generator, we use the content loss defined as the per pixel difference between y and $\hat{y}$ computed using L1 distance as

$$L_g = ||y - \hat{y}||_1 \tag{3}$$

Combining Eq. 1-3 using scaling factors β and μ for L_t and L_c, respectively, the final objective function is given as

$$\min_{G} \max_{D1,D2} [\beta L_t(G, D1) + \mu L_c(G, D2) + L_g] \tag{4}$$

Other network architectures

U-Net: U-Net is a fully convolutional encoder-decoder neural network initially proposed for medical image segmentation [1]. The encoder performs convolution operations that reduce the spatial dimensions of the feature maps while increasing their depth and encoding increasingly abstract representations of the input. The decoder layers also perform convolution operations to restore the spatial size of the features. Skip connections between the encoder and decoder layers are a fundamental network element of U-Net for information propagation from encoder to decoder and stable training of the network. The U-Net architecture used to generate the results in this study is the same as the generator network of P-GAN and is trained using the same hyperparameters using L1-loss function.

GAN: The original GAN framework introduced by Goodfellow *et al.* [2] used generator and discriminator networks with competing losses. Since its introduction, many different network architectures have been proposed, including those that replaced the generator with a deep residual network with upsample blocks (SRGAN) [3], or used U-Net [1]. Here, we used a U-Net based generator (same network architecture as the generator, G in P-GAN, **Supplementary Fig. 2**) and a CNN classifier as discriminator (same network architecture as the CNN discriminator, D2 in P-GAN, **Supplementary Fig. 2**) for cellular recovery.

Pix2Pix: Because conditional GAN [4] was introduced for image-to-image translation and semantic segmentation, its generator also uses U-Net. The network was set up to transform images from the source to the target domain in a way that the generated images cannot be distinguished from the real images of the target domain. Here, a PatchGAN discriminator was adversarially trained to do as well as possible to distinguish the generated fakes.

CycleGAN: This unsupervised strategy for image-to-image translation using unpaired images from the source (speckled images) and the target (averaged images) domain consisted of two GANs that were each trained to transfer images from one domain into another [5]. Each generator took images from its respective domain and generated images of the opposite. While each discriminator was trained to distinguish generated images from real ones, the generators in turn were trained to fool the discriminators. To ensure true style transfer, a cycle consistency prior was enforced whereby the generated images were provided into the generators of the corresponding domain and the result must be identical to the original image used to create the generated image.

MedGAN: Medical image translation using GAN (MedGAN) [6] is an image translation method for medical images that builds upon the recent advances of GANs. MedGAN presented a generator architecture called CasNet that uses multiple U-Nets cascaded together to progressively refine the generated images. In this study we used three U-Nets in the CasNet generator. MedGAN also uses the discriminator network as a trainable feature extractor which penalized the discrepancy between generated and ground truth images.

UP-GAN: Uncertainty guided progressive GAN (UP-GAN) [7] uses an uncertainty guided progressive learning strategy using GAN for medical image translation. By incorporating uncertainty as attention maps, multiple GANs were trained in progressive manner to generate images with increasing image fidelity. We used two successive GANs to recover the RPE images from the input speckled ones. The GANs were trained using an adaptive fidelity loss and adversarial loss. The details of the training parameters are presented in **Supplementary Table 4**.

Validation metrics

The four deep learning based objective image quality assessment metrics used for quantitative evaluation were DISTS [8], PieAPP [9], and LPIPS [10], and FID [11]. DISTS computes the textural and structural similarity between the reference (averaged images) and the recovered images using deep features from five layers of the VGG16 network. PieAPP uses a learning-based method to predict the perceptual error between the averaged and the recovered images. LPIPS provides the human perceptual scores of similarities between the averaged and the recovered images as the L2 distance between the unit-normalized and scaled features of the images extracted from layers of the VGG network. FID uses the pretrained InceptionV3 to estimate the distance between the distribution of the generated images using GANs and the real images. Mean squared error (MSE), peak signal to noise ratio (PSNR), and structural similarity index measure (SSIM) [12] are commonly used metrics for image comparison. However, we chose to use the deep learning-based measures, which demonstrate better performance in quantifying subtle differences in cellular structures resolved with AO-OCT.

Supplementary Figures

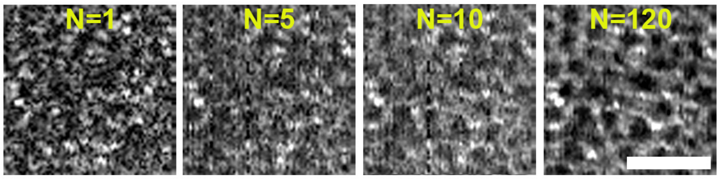

Supplementary Fig. 1. Averaging improves visualization of cellular features. The retinal pigment epithelial (RPE) cell mosaic visualized by averaging increasing numbers (N) of sequentially acquired AO-OCT volumes at sufficiently spaced time intervals. Images with fewer averaged volumes (N=1, 5, or 10) are dominated by speckle noise, obscuring the visibility of RPE cellular structures. Averaging 120 volumes results in visualization of individual RPE cells (dark areas correspond to RPE cell centers). Scale bar: 50 µm.

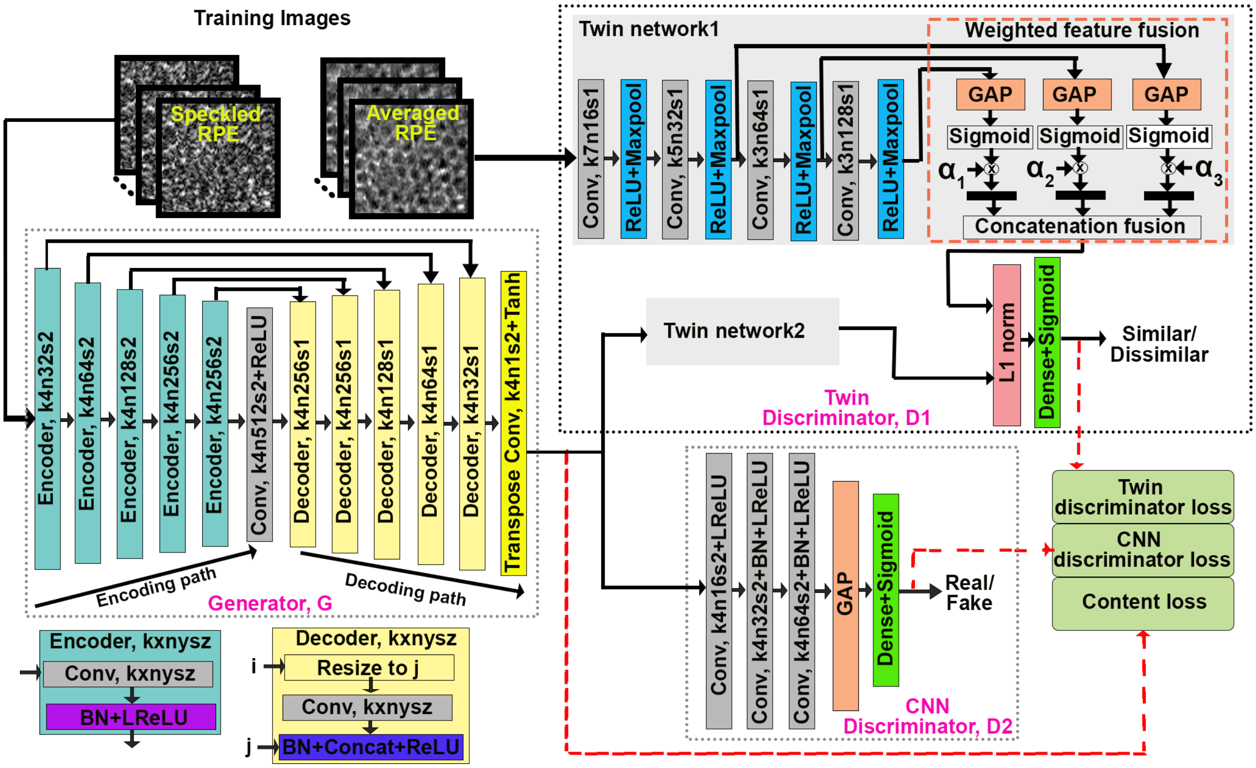

Supplementary Fig. 2. Network architecture of parallel discriminator generative adversarial network (P-GAN). The three neural networks in P-GAN are the generator (G), twin discriminator (D1), and CNN discriminator (D2). G takes as an input single speckled image and creates an image of the RPE using a series of encoders and decoders. The encoding path of G has a series of encoder blocks comprised of a convolution layer (Conv) and batch normalization (BN) with leaky rectified linear unit (LReLU) activation. k, n, and s in the encoder and decoder blocks denote kernel size, number of filters and stride of the convolution. x, y, and z are placeholders for numerical values relating to k, n, and s. The decoding path comprise a series of decoder blocks that perform nearest neighbor interpolation, convolution, BN, and concatenation (shown as skip connection) with the corresponding encoder block output and LReLU. i and j in the decoder block denotes the input from the previous layer and the skip connection, respectively. The last layer of the generator consists of a transpose convolution (Transpose Conv) layer with a Tanh activation. D1 comprises of two identical convolutional neural network (CNN) based twin networks that extract features from the generator created (recovered RPE) and ground truth (averaged images). The Conv layers in the twin networks are followed by rectified linear unit (ReLU) activation and max pooling (Maxpool) operation by a factor of 2. Features from the last three layers are first summarized using global average pooling (GAP) and concatenated with weights (α_1, α_2, and α_3) in the weighted feature fusion (WFF) block and compared using L1 distance. A dense layer with sigmoid activation provides the probabilistic score of similarity between the recovered RPE and the ground truth images. To further ensure perceptual closeness, D2 assigns labels of fake/real to the generator created and averaged images using a sequence of three convolutional layers (each of which is followed by BN (except for the first layer) and LReLU activation) followed by GAP, dense and sigmoid activation. The twin discriminator loss, CNN discriminator loss and the content loss functions ensures proper training of P-GAN. More details on the network and training are provided in **P-GAN network architecture** and **Objective loss functions** section **in Supplementary Methods.**

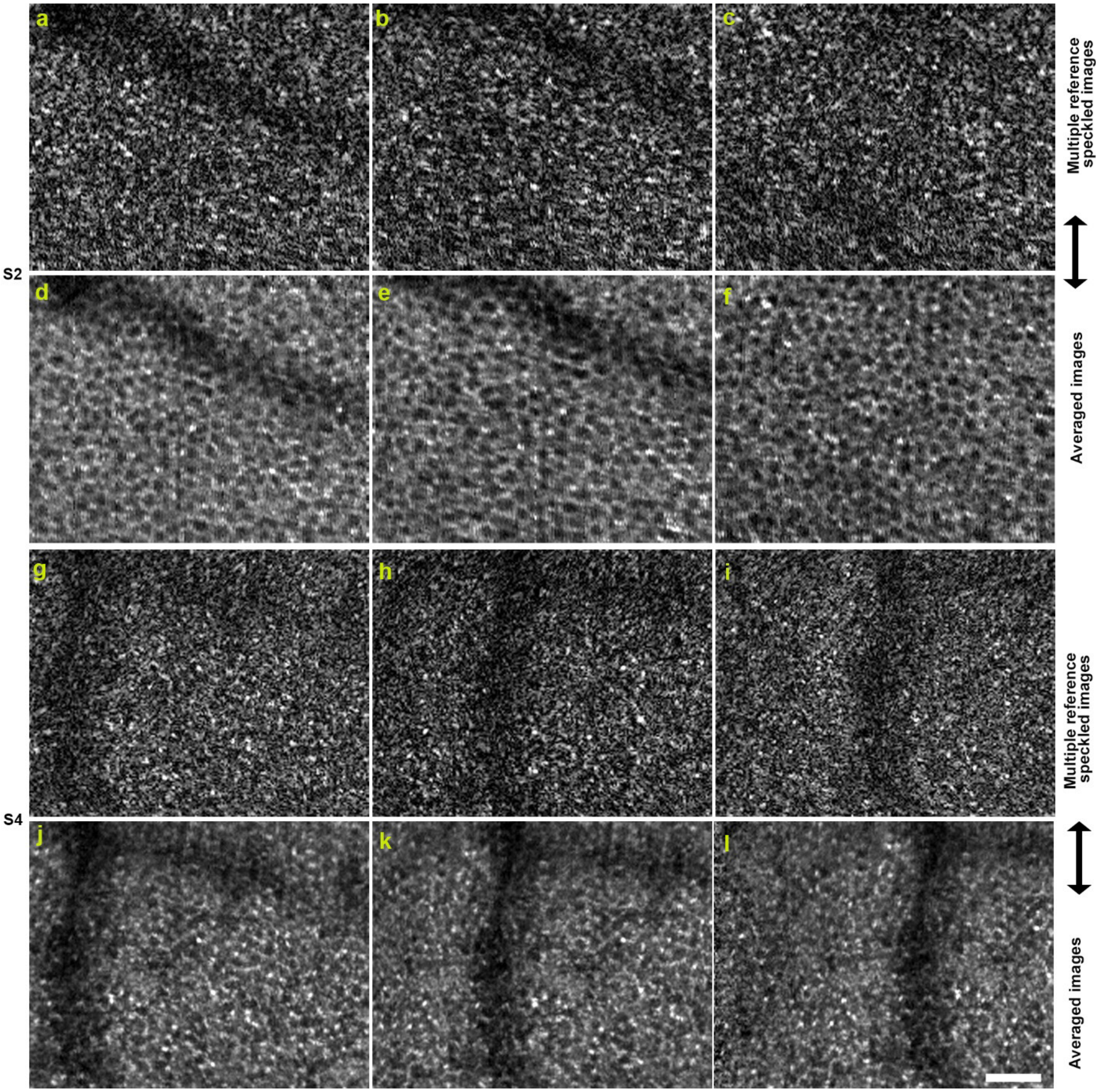

Supplementary Fig. 3. Data augmentation by leveraging natural eye motion. Three examples of averaged images (d-f and j-l) from two participants (S2 and S4) obtained by choosing different reference speckled images (a-c and g-i) for registration. By choosing different reference speckled images for registration, shifted versions of the averaged images were created to augment the training dataset. Scale bar: 50 µm.

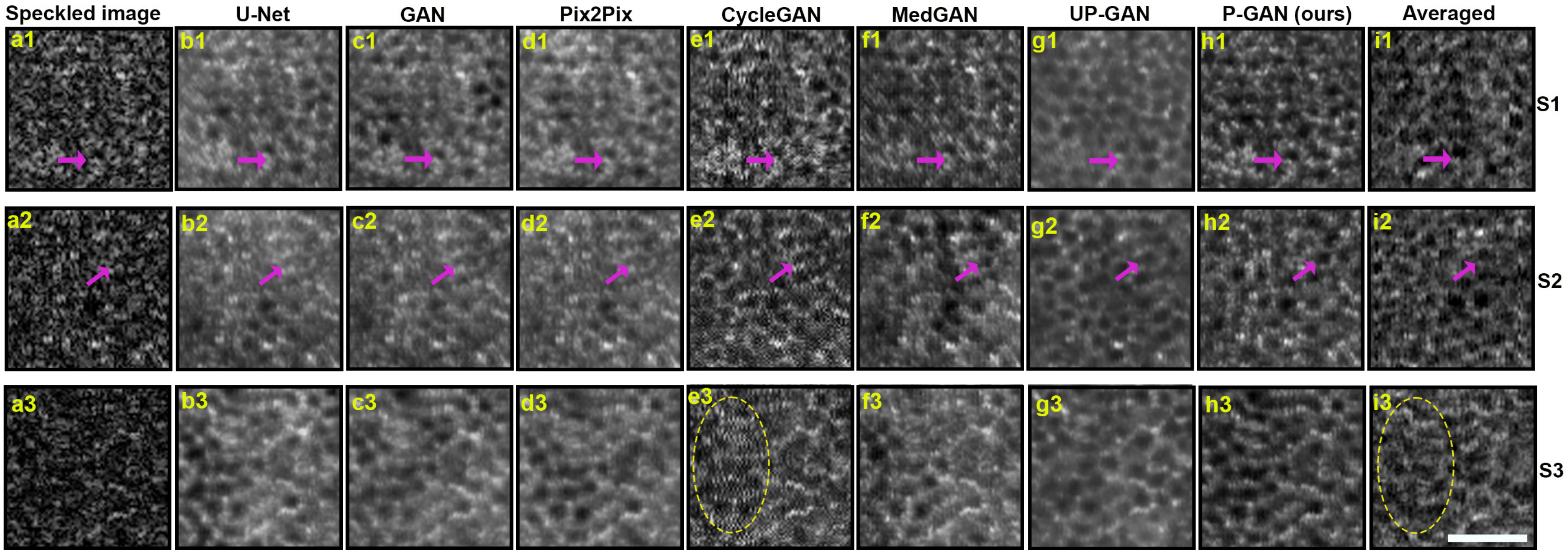

Supplementary Fig. 4. AI recovers cellular structure from a single speckled adaptive optics optical coherence tomography (AO-OCT) image. **(a1-a3)** Example speckled AO-OCT images of RPE acquired from three participants (S1, S2, and S3) obtained from **(b1-b3)** U-Net, **(c1-c3)** generative adversarial network (GAN), **(d1-d3)** Pix2Pix, **(e1-e3)** CycleGAN, **(f1-f3)** medical image translation using GAN (MedGAN), **(g1-g3)** uncertainty guided progressive GAN (UP-GAN), **(h1-h3)** parallel discriminator generative adversarial network (P-GAN) (ours), and **(i1-i3)** by averaging 120 volumes (ground truth). The yellow circle shows a vertical striped artifact in CycleGAN recovered that is not present in the averaged image, for comparison. The magenta arrows show regions where the individual cells are challenging to distinguish in existing methods but can be easily visualized in the P-GAN recovered images. Scale bar: 50 μm.

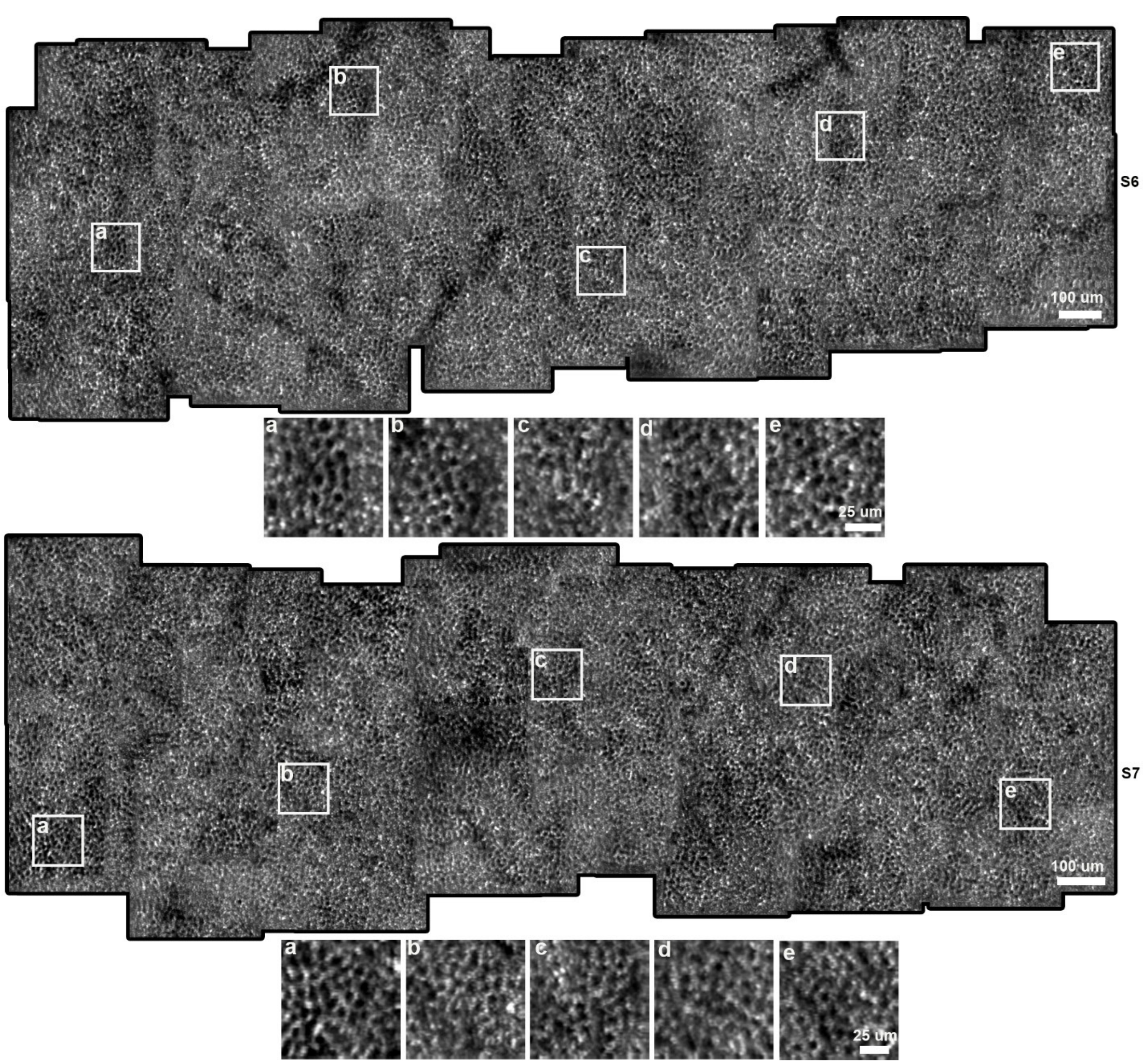

Supplementary Fig. 5. Additional examples of artificial intelligence (AI) derived retinal pigment epithelial (RPE) cell montages from two participants. Visualization of RPE mosaic using parallel discriminator generative adversarial network (P-GAN) recovered images (montages were manually constructed from up to 63 overlapping recovered RPE images of participants S6 and S7). The white squares (a-e) in the montages indicate regions of interest which are further zoomed for visualization purposes. (a-e) for participant S6 correspond to regions of interest at retinal locations 0.1, 1.0, 1.7, 2.3, and 2.8 mm temporal to the fovea, respectively. For participant S7, (a-e) indicate regions of interest at retinal locations 0.1, 0.8, 1.3, 2.0, and 2.6 mm temporal to the fovea, respectively.

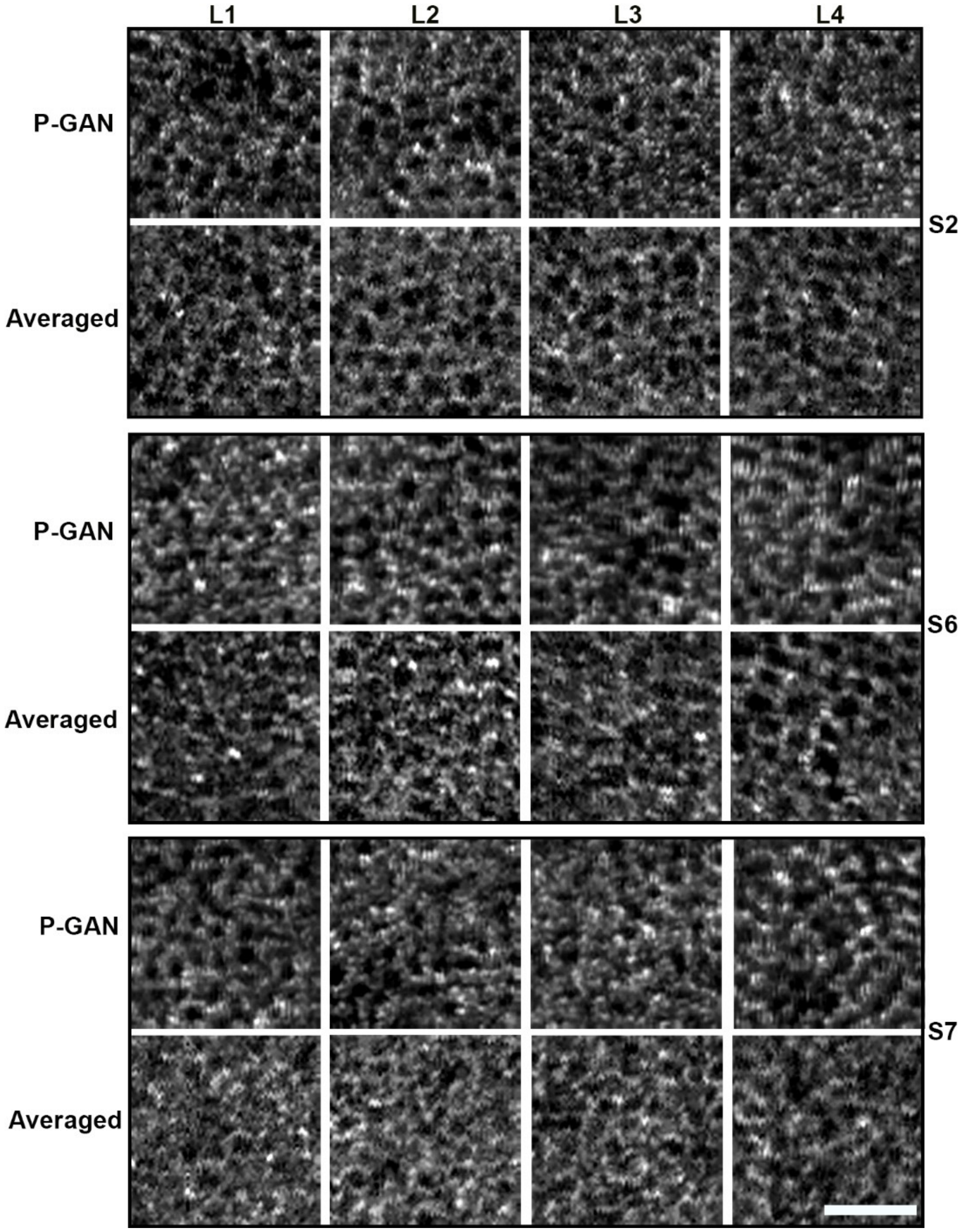

Supplementary Fig. 6. Parallel discriminator generative adversarial network (P-GAN) and averaged (ground truth) images of the retinal pigment epithelial (RPE) cells have similar appearance for experimental data at four different retinal locations. The P-GAN recovered and averaged images at four retinal locations (L1, L2, L3, and L4) of three participants (S2, S6, and S7) that have not been used in training have similar visualization of the cells. L1, L2, L3, and L4 denote regions on the retina located at 0.9, 1.5, 2.1, and 2.7 mm temporal to the fovea, respectively. Scale bar: 50 μm.

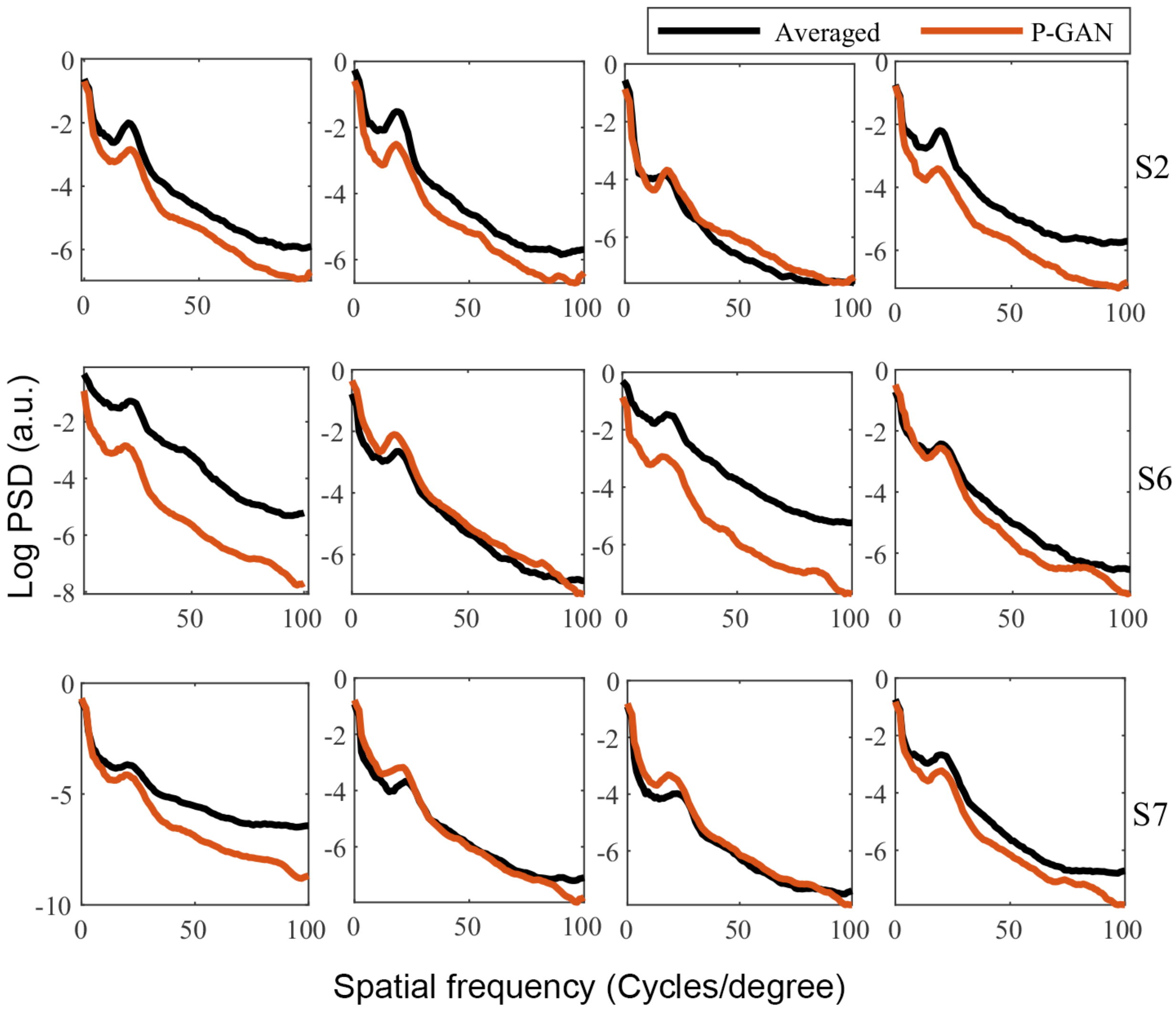

Supplementary Fig. 7. Parallel discriminator generative adversarial network (P-GAN) and averaged images show comparable peaks in the circumferentially averaged power spectral density (PSD). The circumferentially averaged PSD of P-GAN recovered and averaged images at four retinal locations of three participants (S2, S6, and S7) have comparable peak locations (indicative of the cell spacing) for most of the images.

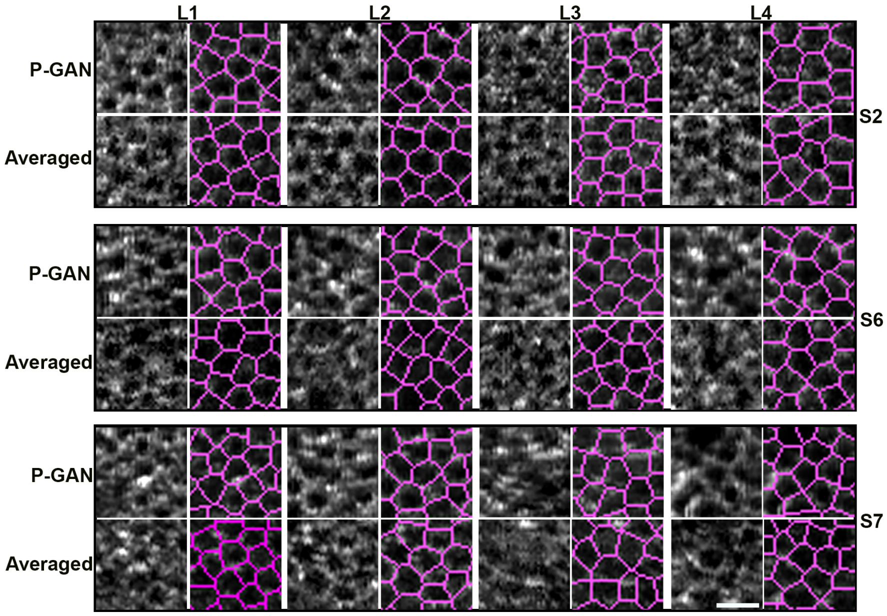

Supplementary Fig. 8. Parallel discriminator generative adversarial network (P-GAN) and averaged (ground truth) images show similar packing properties of RPE cells. Voronoi analysis of P-GAN recovered and averaged images at four retinal locations (L1, L2, L3, and L4) of three participants (S2, S6, and S7) reveal the retinal pigment epithelial (RPE) cells. L1, L2, L3, and L4 denote regions on the retina located at 0.9, 1.5, 2.1, and 2.7 mm temporal to the fovea, respectively. Scale bar: 25 μm.

Supplementary Tables

Supplementary Table 1. Participant information

Participant ID	Eye†	No. of Imaging locations‡	Training and testing of the AI model	Experimental validation
S1	OD	3	Yes	No
S2	OD	2	Yes	No
	OS	63	No	Yes
S3	OD	4	Yes	No
S4	OD	3	Yes	No
S5	OD	4	Yes	No
S6	OS	63	No	Yes
S7	OS	63	No	Yes

†OD = right eye, OS = left eye

‡All locations acquired temporal to the fovea

Supplementary Table 2. Effect of fusing features from different layers of the twin CNN on RPE recovery

**Configuration**	**DISTS** (↓)	**LPIPS** (↓)	**PieAPP** (↓)	**FID** (↓)
No fusion	0.22	0.38	0.72	166.44
Fusion of last two layers	0.21	0.38	**0.69**	155.18
Fusion of last three layers	**0.20**	**0.37**	0.78	**142.60**
Fusion of all four layers	0.21	0.38	0.75	142.65

DISTS-deep image structure and texture similarity [8]; PieAPP-perceptual image error assessment through pairwise preference [9]; LPIPS-learned perceptual image patch similarity [10]; FID-Fréchet Inception Distance [11]. The arrow (↓) indicates that lower scores are better. To find the appropriate number of twin CNN layers to fuse, we investigated the performance resulting from no fusion (using the output from the last layer of twin CNN for similarity assessment on the twin discriminator), compared to fusing the last two, three, or all four convolutional layers of the twin CNN with equal weight value of one. The best performance is shown in bold. The fusion of three layers provides best performance across most of the measures. All layers are fused with equal weight value of one.

Supplementary Table 3. Effect of the choice of the weight in the weighted feature fusion (WFF) block of P-GAN on the performance

Weight configuration	DISTS	LPIPS	PieAPP	FID
Learnable weights	0.21	0.39	0.71	145.74
$\alpha_1 = \alpha_2 = \alpha_3 = 1$	0.20	0.37	0.78	142.60
$\alpha_1 = 1, \alpha_2 = \alpha_3 = 0.1$	0.27	0.44	0.70	**124.73**
$\alpha_1 = 1, \alpha_2 = \alpha_3 = 0.2$	**0.19**	**0.36**	**0.66**	139.62
$\alpha_1 = 1, \alpha_2 = \alpha_3 = 0.3$	0.23	0.37	0.78	151.77
$\alpha_1 = 1, \alpha_2 = \alpha_3 = 0.4$	0.21	0.36	0.87	147.17
$\alpha_1 = 1, \alpha_2 = \alpha_3 = 0.5$	0.22	0.39	0.78	218.99
$\alpha_1 = 1, \alpha_2 = \alpha_3 = 0.6$	0.22	0.39	0.75	152.16
$\alpha_1 = 1, \alpha_2 = \alpha_3 = 0.7$	0.20	0.36	0.70	154.37
$\alpha_1 = 1, \alpha_2 = \alpha_3 = 0.8$	0.21	0.37	0.74	156.35
$\alpha_1 = 1, \alpha_2 = \alpha_3 = 0.9$	0.21	0.38	0.69	157.12

In order to find the appropriate weights for feature fusion of the last three layers, different weight selection strategies were tested: (i) learnable weighting scheme, where the weight parameters are automatically learned along with the other model parameters, (ii) fixed parameters equal weighs, where the weight parameters are set to a constant value of 1, and (iii) fixed parameters unequal weights, where we varied the weights of the intermediate layers from 0-0.9 with steps of 0.1 while keeping the weights of the last convolutional layer fixed to 1. The recovery performance shows that the weight combination of $\alpha_1 = 1, \alpha_2 = \alpha_3 = 0.2$, provided the best overall performance (shown in bold) for the majority of the metrics.

Supplementary Table 4. Hyper parameters

Parameter	U-Net, GAN, Pix2Pix, CycleGAN and P-GAN	MedGAN	UP-GAN
Training image size	150 × 150 pixels	150 × 150 pixels	150 × 150 pixels
No. of paired training images	5968	5968	5968
Batch size	8	8	8
No. of epochs	100	50	50
Learning rate	0.0002	0.0002	0.00001
Optimizer	Adam	Adam	Adam

Supplementary Table 5. Comparison of cellular recovery performance across five participants

Method	DISTS (↓)	PieAPP (↓)	LPIPS (↓)	FID (↓)
U-Net	0.25 ± 0.01	1.38 ± 0.31	0.42 ± 0.03	175.13 ± 31.2
GAN	0.24 ± 0.01	1.29 ± 0.24	0.41 ± 0.02	161.56 ± 10.4
Pix2Pix	0.24 ± 0.02	1.31 ± 0.29	0.42 ± 0.04	172.83 ± 30.3
CycleGAN	0.26 ± 0.02	1.13 ± 0.30	0.46 ± 0.03	151.56 ± 40.0
MedGAN	0.23 ± 0.02	1.04 ± 0.23	0.43 ± 0.01	173.44 ± 40.8
UP-GAN	0.23 ± 0.02	1.49 ± 0.33	0.42 ± 0.03	176.44 ± 40.0
**P-GAN (ours)**	**0.20 ± 0.01**	**0.94 ± 0.17**	**0.38 ± 0.02**	**150.07 ± 22.1**

The arrow (↓) indicates that lower scores are better. P-GAN (shown in bold) had the highest perceptual similarity between recovered images compared to ground truth averaged images across these networks. All values expressed as mean ± SD.

Supplementary Table 6. Effect of eye motion based data augmentation on cell recovery performance

Dataset	No. of paired training examples	DISTS	LPIPS	PieAPP	FID
Initial dataset†	136	0.25	0.45	1.16	216.11
Augmented dataset‡ (leveraging eye motion-based data augmentation)	5996	0.20	0.38	0.94	150.07

† The initial dataset was created by extracting pairs of speckled and averaged images from 17 locations. The 17 paired images were cropped (4 crops per image of size 150 × 150 pixels) and horizontally flipped to obtain 17×4×2=136 image patch pairs for training the network.

‡ Leveraging the natural eye motion of the participants, we could get 40-50 paired images from the 17 locations. These images were cropped (4 crops per image of size 150 × 150 pixels) to obtain 2998 image patch pairs which were further augmented using horizontal flipping to create a dataset of 5996 image pairs for training the network. Using the augmented dataset, a 44-fold increase in data was achieved with a corresponding improvement in all the objective metrics.

Supplementary Table 7. Recovery performance across four retinal locations from three participants

DISTS	PieAPP	LPIPS	FID
0.23 ± 0.02	0.82 ± 0.05	0.44 ± 0.04	206.0 ± 22.7

The objective scores for the recovery performance for the experimental data are comparable to the test data (**Supplementary Table 5**). All values given as mean ± SD.

Supplementary Table 8. RPE cell spacing and peak distinctiveness of three participants at four retinal locations estimated from power spectrum analysis

Participant ID	Imaging location†	RPE spacing (µm)		Spacing error (µm)	Peak distinctiveness (a.u.)	
		Averaged‡	P-GAN recovered		Averaged	P-GAN recovered
S2	L1	14.8	14.0	0.8	0.62	0.39
	L2	15.6	15.6	0.0	0.58	0.63
	L3	16.5	15.6	0.9	0.14	0.69
	L4	14.8	15.6	-0.8	0.56	0.37
S6	L1	12.8	14.0	-1.2	0.25	0.27
	L2	14.0	15.6	-1.6	0.33	0.56
	L3	14.8	16.5	-1.7	0.32	0.28
	L4	14.8	14.8	0.0	0.27	0.34
S7	L1	14.0	14.0	0.0	0.17	0.25
	L2	12.7	13.4	-0.7	0.37	0.24
	L3	12.7	15.6	-2.9	0.12	0.37
	L4	14.0	14.0	0.0	0.30	0.36

† L1, L2, L3, and L4 denote 0.5 × 0.5 mm regions of interest imaged at 0.9, 1.5, 2.1, and 2.7 mm temporal to the fovea, respectively.

‡ The averaged images are obtained by averaging 120 AO-OCT volumes.

Supplementary Table 9: RPE cell spacing of three participants at four locations estimated from Voronoi analysis

Participant ID	Imaging location†	RPE spacing (μm)		Spacing error (μm)
		Averaged‡	P-GAN recovered	
S2	L1	14.7	14.1	0.6
	L2	15.2	14.1	1.1
	L3	15.2	14.8	0.4
	L4	15.5	14.3	1.2
S6	L1	14.4	14.7	-0.3
	L2	16.9	15.1	1.8
	L3	14.8	14.4	0.4
	L4	15.1	16.7	-1.6
S7	L1	14.0	13.6	0.4
	L2	14.0	14.1	-0.1
	L3	15.2	14.8	0.4
	L4	15.5	14.3	1.2

† L1, L2, L3, and L4 denote 0.5 × 0.5 mm regions of interest imaged at 0.9, 1.5, 2.1, and 2.7 mm temporal to the fovea, respectively.

‡ The averaged images are obtained by averaging 120 AO-OCT volumes.

Supplementary Table 10. Network characteristics and times

Characteristic	U-Net	GAN	Pix2Pix	CycleGAN	MedGAN	UP-GAN	P-GAN (ours)
No. of parameters (millions)	9.41	9.45	9.45	84.45	79.92	28.90	9.56
Training time (hours)	5.3	5.5	5.6	32	24	4.5	6.8
Test image size (pixels)	300 × 200	300 × 200	300 × 200	300 × 200	300 × 200	300 × 200	300 × 200
Test time (seconds)	0.1 s	0.1 s	0.1 s	0.5 s	0.26 s	1.41 s	0.1 s

Supplementary Table 11. Comparison of AO-OCT imaging and AI enabled AO-OCT imaging

Parameters	AO-OCT imaging [13]	AI enabled AO-OCT imaging
Image acquisition time†	6.3 h	30 min
No. of volumes acquired per location	120	10*
Data processing time‡	13 days	2.7 h
Image registration	Yes	No
Acquired data file size	2.8 TB	0.23 TB

† It takes 6 minutes to acquire 120 volumes from one retinal location using AO-OCT. To image 63 locations (covering 1 mm × 3 mm of the retina in this paper), it would take 6.3 h (6 minutes × 63) compared to only 30 minutes using AI enabled AO-OCT imaging.

*A total of 10 volumes were acquired at each location from which the one with least distortion (subjectively-determined minimal motion artifacts and no eye blinks) was selected as input to the P-GAN for cellular recovery.

‡ The data processing time for [13] includes AO-OCT reconstruction (converting raw data to volumes (120 volumes per location × 63 locations)), registration to correct for eye motion, and averaging to suppress noise and increase the cellular contrast. Combined, the data processing time is 4.8 hours for each location with 120 volumes. The estimated data processing time required for AO-OCT data acquired from 63 locations is ~13 days (4.8 h × 63 = 302.4 h). For AI enabled AO-OCT imaging we acquire only 10 volumes per locations and also there is no requirement of registration and averaging. Hence, to convert the raw data to AO-OCT volumes, it takes 155 seconds for 10 volumes at each location and for 63 locations, it takes only 2.7 hours (155 s × 63). AI based cell recovery time is neglected as it is small (0.1 s) compared to the imaging and processing times.

Altogether the total time taken for conventional imaging is 6.3 h (imaging) + 13 days (data processing) = 318.3 h. The time taken by AI enabled imaging is 30 min (imaging) + 2.7 h (processing) = 3.2 h. Therefore, a 99.4 (318.3/3.2) fold improvement is achieved using AI.

Supplementary References

1. Ronneberger, O., P. Fischer, and T. Brox. *U-net: Convolutional networks for biomedical image segmentation*. in *International Conference on Medical image computing and computer-assisted intervention*. 2015. p. 234-241.
2. Goodfellow, I., et al., *Generative adversarial networks.* Communications of the ACM, 2020. **63**(11): p. 139-144.
3. Ledig, C., et al. *Photo-realistic single image super-resolution using a generative adversarial network*. in *Proceedings of the IEEE conference on computer vision and pattern recognition*. 2017. p . 4681-4690.
4. Isola, P., et al. *Image-to-image translation with conditional adversarial networks*. in *Proceedings of the IEEE conference on computer vision and pattern recognition*. 2017. P. 1125-1134.
5. Zhu, J.-Y., et al. *Unpaired image-to-image translation using cycle-consistent adversarial networks*. in *Proceedings of the IEEE international conference on computer vision*. 2017. p. 1113-2232.
6. Armanious, K., et al., *MedGAN: Medical image translation using GANs.* Computerized medical imaging and graphics, 2020. **79**: p. 101684.
7. Upadhyay, U., et al. *Uncertainty-guided progressive GANs for medical image translation*. in *Medical Image Computing and Computer Assisted Intervention–MICCAI 2021: 24th International Conference, Strasbourg, France, September 27–October 1, 2021, Proceedings, Part III 24*. 2021. p. 614-624.
8. Ding, K., et al., *Image quality assessment: Unifying structure and texture similarity.* IEEE transactions on pattern analysis and machine intelligence, 2020. 44(5), p. 2567-2581.
9. Prashnani, E., et al. *Pieapp: Perceptual image-error assessment through pairwise preference*. in *Proceedings of the IEEE Conference on Computer Vision and Pattern Recognition*. 2018. p. 1808-1817.
10. Zhang, R., et al. *The unreasonable effectiveness of deep features as a perceptual metric*. in *Proceedings of the IEEE conference on computer vision and pattern recognition*. 2018. p. 586-595.
11. Heusel, M., et al., *GANs trained by a two time-scale update rule converge to a local nash equilibrium.* Advances in neural information processing systems, 2017. **30**.
12. Wang, Z., et al., *Image quality assessment: from error visibility to structural similarity.* IEEE transactions on image processing, 2004. **13**(4): p. 600-612.
13. Bower, A.J., et al., *Integrating adaptive optics-SLO and OCT for multimodal visualization of the human retinal pigment epithelial mosaic.* Biomedical Optics Express, 2021. **12**(3): p. 1449-1466.
