# Supplementary material for: Revealing speckle obscured living human retinal cells with artificial intelligence assisted adaptive optics optical coherence tomography
Source: Commun Med (Lond). 2024 Apr 10;4:68. doi: 10.1038/s43856-024-00483-1 (PMC11006674; doi:10.1038/s43856-024-00483-1)
Supplement: Supplementary file 2 — Description of Additional Supplementary Files [file 43856_2024_483_MOESM2_ESM.pdf]

Description of Additional Supplementary Files

File Name: Supplementary Data 1
Description: Numerical source data used to create Fig. 2 g-i, Fig. 3 j, and Fig. 5 in the manuscript.

File Name: Supplementary Movie 1
Description: P-GAN recovers the retinal pigment epithelial (RPE) cells from speckled AO-OCT enface images. Toggling between speckled and P-GAN recovered RPE image for participant S1 shows that P-GAN suppressed the noise and revealed the RPE cellular structure. Scale bar: 50 µm.

File Name: Supplementary Movie 2
Description: Comparison between P-GAN recovered RPE image and ground truth averaged RPE image. Toggling between P-GAN recovered and averaged RPE images for participant S2 shows similarity in the cells. Scale bar: 50 µm.

File Name: Supplementary Movie 3
Description: Comparison of GAN recovered RPE images and ground truth averaged RPE images. Video shows simultaneous visual comparison of the speckled RPE, GAN, Pix2Pix, CycleGAN, P-GAN recovered RPE, and averaged ground truth images for three participants (S1-S3). Scale bar: 50 µm.
