# Supplementary material for: Revealing speckle obscured living human retinal cells with artificial intelligence assisted adaptive optics optical coherence tomography
Source: Commun Med (Lond). 2024 Apr 10;4:68. doi: 10.1038/s43856-024-00483-1 (PMC11006674; doi:10.1038/s43856-024-00483-1)
Supplement: Supplementary file 7 — Reporting Summary [file 43856_2024_483_MOESM7_ESM.pdf]

Corresponding author(s): Johnny Tam

Last updated by author(s): 1/4/2024

Reporting Summary

Nature Portfolio wishes to improve the reproducibility of the work that we publish. This form provides structure for consistency and transparency in reporting. For further information on Nature Portfolio policies, see our Editorial Policies and the Editorial Policy Checklist.

Statistics

For all statistical analyses, confirm that the following items are present in the figure legend, table legend, main text, or Methods section.

n/a	Confirmed	
☐	☒	The exact sample size (*n*) for each experimental group/condition, given as a discrete number and unit of measurement
☐	☒	A statement on whether measurements were taken from distinct samples or whether the same sample was measured repeatedly
☒	☐	The statistical test(s) used AND whether they are one- or two-sided<br>*Only common tests should be described solely by name; describe more complex techniques in the Methods section.*
☒	☐	A description of all covariates tested
☒	☐	A description of any assumptions or corrections, such as tests of normality and adjustment for multiple comparisons
☐	☒	A full description of the statistical parameters including central tendency (e.g. means) or other basic estimates (e.g. regression coefficient) AND variation (e.g. standard deviation) or associated estimates of uncertainty (e.g. confidence intervals)
☒	☐	For null hypothesis testing, the test statistic (e.g. *F*, *t*, *r*) with confidence intervals, effect sizes, degrees of freedom and *P* value noted<br>*Give P values as exact values whenever suitable.*
☒	☐	For Bayesian analysis, information on the choice of priors and Markov chain Monte Carlo settings
☒	☐	For hierarchical and complex designs, identification of the appropriate level for tests and full reporting of outcomes
☒	☐	Estimates of effect sizes (e.g. Cohen's *d*, Pearson's *r*), indicating how they were calculated

Our web collection on statistics for biologists contains articles on many of the points above.

Software and code

Policy information about availability of computer code

Data collection	Data collection was performed using a custom retinal imager that has been previously reported (ref 13).
Data analysis	MATLAB, Python, Adobe Photoshop, and Microsoft Excel were used for data visualization and analysis.

For manuscripts utilizing custom algorithms or software that are central to the research but not yet described in published literature, software must be made available to editors and reviewers. We strongly encourage code deposition in a community repository (e.g. GitHub). See the Nature Portfolio guidelines for submitting code & software for further information.

Data

Policy information about availability of data

All manuscripts must include a data availability statement. This statement should provide the following information, where applicable:

- Accession codes, unique identifiers, or web links for publicly available datasets
- A description of any restrictions on data availability
- For clinical datasets or third party data, please ensure that the statement adheres to our policy

All data are available in the main text or the supplementary materials.

Human research participants

Policy information about studies involving human research participants and Sex and Gender in Research.

Reporting on sex and gender	The age of the participants was 29.1 +/- 9.1 years with 6 females and 1 male.
Population characteristics	The participants of the study included subjects with no signs of ocular diseases.
Recruitment	Subjects were recruited from the National Eye Institute Eye Clinic at the National Institutes of Health, Bethesda, Maryland. Informed consent was obtained from all participants prior to enrollment. Subjects were included in the study if they had no signs of ocular disease after a comprehensive dilated eye exam.
Ethics oversight	This study was approved by the institutional review board of the National Institutes of Health and was conducted in accordance with the Declaration of Helsinki.

Note that full information on the approval of the study protocol must also be provided in the manuscript.

Field-specific reporting

Please select the one below that is the best fit for your research. If you are not sure, read the appropriate sections before making your selection.

☒ Life sciences ☐ Behavioural & social sciences ☐ Ecological, evolutionary & environmental sciences

For a reference copy of the document with all sections, see nature.com/documents/nr-reporting-summary-flat.pdf

Life sciences study design

All studies must disclose on these points even when the disclosure is negative.

Sample size	As this study presented a new method for retinal pigment epithelial cells visualization, rather than an attempt to illustrate a statistically significant biological phenomenon, no sample size considerations were relevant. That said, the number of subjects included was larger than the typical sample sizes for adaptive optics optical coherence tomography imaging studies.
Data exclusions	Since adaptive optics video sequences were captured, we excluded any raw data captured during blinks, excessive eye motion, or other drop in image quality that prevented image sequences from being registered to a reference frame, which is the standard approach for adaptive optics retinal imaging data.
Replication	The proposed technique was tested on seven subjects of varied age groups with adaptive optics optical coherence tomography imaging performed at different retinal locations. The designed method was successful in enabling the visualization of retinal pigment epithelial cells for all seven subjects used in the study.
Randomization	Randomization was not applicable. A leave one subject out based validation was adopted where the performance of the neural network was validated on data that was not seen during the network training phase.
Blinding	Blinding was not applicable, since no anticipated result was expected.

Reporting for specific materials, systems and methods

We require information from authors about some types of materials, experimental systems and methods used in many studies. Here, indicate whether each material, system or method listed is relevant to your study. If you are not sure if a list item applies to your research, read the appropriate section before selecting a response.

Materials & experimental systems

n/a	Involved in the study	
☒	☐	Antibodies
☒	☐	Eukaryotic cell lines
☒	☐	Palaeontology and archaeology
☒	☐	Animals and other organisms
☒	☐	Clinical data
☒	☐	Dual use research of concern

Methods

n/a	Involved in the study	
☒	☐	ChIP-seq
☒	☐	Flow cytometry
☒	☐	MRI-based neuroimaging
